# Investigating Saccade-Onset Locked EEG Signatures of Face Perception during Free-Viewing in a Naturalistic Virtual Environment

**DOI:** 10.1523/ENEURO.0573-24.2025

**Published:** 2025-08-29

**Authors:** Debora Nolte, Vincent Schmidt, Aitana Grasso-Cladera, Peter König

**Affiliations:** ^1^Institute of Cognitive Science, University of Osnabrück, Osnabrück 49090, Germany; ^2^Department of Neurophysiology and Pathophysiology, University Medical Center Hamburg-Eppendorf, Hamburg 20246, Germany

**Keywords:** face perception, fixation-onset ERP, free-viewing, N170, saccade-onset ERP, virtual reality

## Abstract

Current research strives to investigate cognitive processes under natural conditions. Virtual reality and EEG are promising techniques combining naturalistic settings with close experimental control. However, many questions and technical challenges remain, e.g., are saccade onsets a suitable replacement of fixation onsets as key events in continuous gaze trajectories ( Amme et al., 2024), and consequently, can VR capture differences across different stimulus categories associated with varying saccade durations? To address both questions, we investigate the N170 face effect in humans (14 males, 19 females, zero diverse) using a free-viewing and free-movement immersive VR study that contained houses, various background stimuli, and, notably, static and moving pedestrians to study face perception under naturalistic conditions. Our results show that aligning trials to saccade onsets leads to more well-defined ERPs than fixation onsets, especially for the P100 component, demonstrating that saccade-onset ERPs are a better-suited analysis method for this type of experiment. Furthermore, we observe an evolution of category-based differences, i.e., face versus background saccade-onset ERPs, compatible with previous reports but extending in a large temporal window and including all electrode sites at different points in time. In summary, employing VR, EEG, and eye-tracking to investigate differences across fixation categories provides insights into the relevance of saccadic onsets as event triggers and enhances our understanding of cognitive processes in naturalistic settings.

## Significance Statement

With the effort of investigating and understanding cognitive processes under naturalistic conditions, combining virtual reality and EEG can be fruitful in implementing free-viewing studies. The current work combines these technologies to explore key challenges in the context of face perception in an immersive virtual environment. Our results show that saccade-onset ERPs yield more precise measurements when analyzing continuous eye-tracking data than fixation onsets. Furthermore, when processing face compared with background stimuli, distinct temporal patterns encompassing all electrode sites can be observed, offering new insights into face perception. Overall, this work highlights the potential of integrating VR and EEG to advance our understanding of cognitive processes in naturalistic settings.

## Introduction

In recent years, a step has been taken to study and understand cognitive processes under natural conditions, capturing them in dynamic, real-world environments (Tromp et al., 2018; Shamay-Tsoory and Mendelsohn, 2019; Rounds et al., 2020; Gert et al., 2022; Stangl et al., 2023). A central aspect of this approach are free-viewing paradigms, where subjects move their eyes and actively choose where to direct their gazes (Gert et al., 2022; Amme et al., 2024), allowing us to study the spontaneous and adaptive nature of real-world visual behavior (Shamay-Tsoory and Mendelsohn, 2019; Stangl et al., 2023). For these studies, virtual reality (VR) is emerging as a powerful tool, combining the high experimental control of laboratory setups with free-viewing experiences of real life (Bohil et al., 2011; Pan and Hamilton, 2018; Bell et al., 2020). Supporting the potential of VR, recent studies demonstrated that VR can provide findings similar to real life (Nolte et al., 2025) and is suitable for analyzing eye-tracking data in naturalistic environments (Clay et al., 2019; Llanes-Jurado et al., 2020; Nolte et al., 2024). Beyond eye movements, integrating VR with electroencephalography (EEG) allows for exploring neural responses to naturalistic visual behavior (Tromp et al., 2018; Rounds et al., 2020; Stangl et al., 2023) and measuring fixation-onset event–related potentials (ERPs; Nolte et al., 2024). This highlights the potential of combining VR and EEG to study cognitive processes under naturalistic, free-viewing conditions.

While VR has proven helpful in investigating vision and neural processes under naturalistic conditions, many questions and technical challenges remain. For instance, although neural processes can be studied with VR–EEG setups (Tromp et al., 2018; Rounds et al., 2020; Nolte et al., 2024), the feasibility of using this combination to examine fixation-onset ERP differences across experimental conditions remains to be explored. Notably, a recent magnetoencephalography (MEG) study investigated fixation- and saccade-onset ERP differences during naturalistic viewing of pictures and found saccade-onset ERPs better suited for studying early visual components (Amme et al., 2024). Building on these findings, a question arises: Do saccade onsets provide the optimal alignment for ERP analysis in free-viewing studies? Furthermore, if saccade onsets are the preferred alignment, can VR capture differences across stimulus categories varying in saccade characteristics?

To address the first question, we can assess the timing of a saccade-onset P100 in an immersive free-viewing study. Employing these saccade-onset ERPs to study a well-established effect, such as the N170 face effect (Rossion and Jacques, 2008; Eimer, 2011), can tackle the second question. The N170 effect, described as a stronger ERP response of faces (Rossion and Jacques, 2008; Eimer, 2011) and bodies (Hietanen and Nummenmaa, 2011) compared with other stimuli, has been replicated in free-viewing picture setups (de Lissa et al., 2019; Auerbach-Asch et al., 2020; Gert et al., 2022) and with virtual humans (Wheatley et al., 2011). Thus, the N170 effect is ideal for investigating whether saccade onsets provide superior temporal alignment and whether the ERPs can reveal differences between experimental conditions in an immersive free-viewing study.

The current experiment was designed as a three-dimensional virtual city populated with avatars. Participants *ad libitum* explored the city center while we recorded their eye movements and EEG signals. We explored the temporal alignment of fixation- and saccade-onset ERPs in line with previous research (Amme et al., 2024) to determine the more suitable option. Furthermore, to investigate differences between stimulus categories, we split our data into head, body, and background stimuli (Gert et al., 2022). We hypothesized the highest N170 amplitude for heads, followed by bodies, and the smallest for background stimuli. Our results supported a saccade-onset alignment. Noise-level differences across stimulus categories prevented directly testing the N170 effect; instead, a mass univariate analysis revealed differences between all three categories, partially supporting our hypothesis. Overall, these results underline the suitability of combining EEG with VR but highlight new methodological challenges of free-viewing studies.

## Materials and Methods

### Subjects

Overall, 61 subjects were invited to the lab for the experiment. We could not start the recording for two subjects due to technical difficulties. Out of the remaining 59 subjects, a total of 26 subjects were excluded: 5 quit due to motion sickness, 3 subjects were excluded as they did not follow task instructions and left the central square for >10% of the time, and 18 had to be excluded due to data issues that occurred during or after the recording including 8 subjects that were excluded due to unsynchronized drifts between or within multiple data streams recorded. After applying a conservative approach to data inclusion to maintain high data quality, the final dataset included 33 subjects (19 females, zero diverse; mean age 22.63 ± 2.48 years). All subjects had normal or corrected to normal vision, did not report any neurological disorders, gave written informed consent before participating, and were rewarded with monetary compensation or participation hours. The ethics commission of the University of Osnabrück approved the study.

### Experimental setup

**Figure 1.**
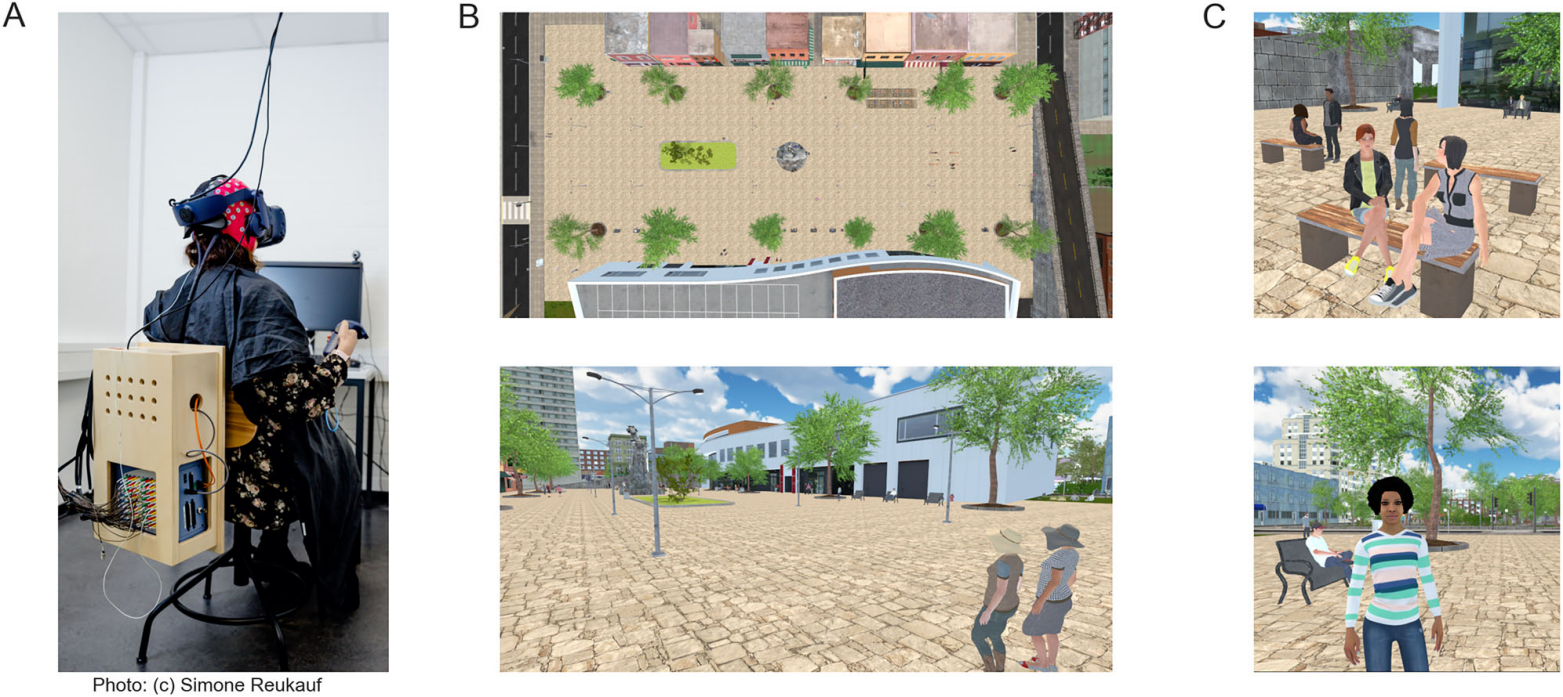
Experimental setup. ***A***, Participants were seated on a swivel chair, wearing an EEG cap and VR glasses. The EEG equipment, specifically the amplifier, was stored on the back of the chair. ***B***, The walkable area was confined to the beige floor and comprised the center of the VR scene. ***C***, Different pedestrians were distributed throughout the city square.

A detailed description of the data and experimental design can be found in previous publications (Nolte et al., 2024, 2025). Below, we provide the essential aspects relevant to the current study. The experiment was developed using Unity3D (Unity Technologies, 2021) version 2019.4.21f1, employing the built-in Universal Render Pipeline/Unlit with one central light source. To maintain perceptual consistency, we minimized shaded areas. The virtual environment was displayed at a constant 90 Hz frame rate via the HTC Vive Pro Eye head-mounted display (HMD; 110° field of view, resolution 1,440 × 1,600 pixels per eye, refresh rate 90 Hz; HTC Corporation, 2018a). The advantage of the HTC Vive Pro Eye HMD is the integrated Tobii eye-tracker (0.5–1.1° accuracy, 110° field of view), allowing us to actively record the subject's eye movements. Eye-tracking was facilitated using the SRanipal SDK (v1.1.0.1; HTC Corporation, 2018b), and spatial tracking was provided by the HTC Vive Lighthouse 2.0 system (HTC Corporation, 2018c). Participants moved within the virtual city using HTC Vive controllers 2.0 (HTC Corporation, 2018d; sensory feedback disabled), with the direction of movement determined by the head orientation. The data were recorded on an Alienware Aurora Ryzen computer (Windows 10, 64-bit, build 19044, 6553 MB RAM; Nvidia RTX 3090 GPU, driver version 31.0.15.2698; AMD Ryzen 9 3900X 12-Core CPU). Simultaneously, EEG data using a 10/20 64-channel Ag/AgCl-electrode system with a Waveguard cap (ANT Neuro) and a Refa8 (TMSi) amplifier were recorded using the OpenVIBE acquisition server (v2.2.0; Renard et al., 2010) on a Dell Precision 5820 Tower (Windows 10, 64 bit, build 19044; Nvidia RTX 2080 Ti GPU, driver version 31.0.15.1694; Intel Xeon W-2133 CPU). The EEG data were collected at 1,024 Hz with an average reference and a ground electrode under the left collarbone. Impedances were kept below 10 kΩ. Synchronization between the EEG and VR systems was achieved using the LabStreamingLayer (LSL; Kothe, 2014). Throughout the experiment, participants were seated on a swivel chair to allow full 360° body rotation (Fig. 1*A*).

### The experimental procedure

The entire experiment lasted 2.5 h. At first arrival, participants filled out informed consent sheets and received instructions about the experiment. Following this, participants underwent a 1 min motion sickness test in the same virtual environment but in an unreachable part of the city. They were instructed to move toward a red sphere at the end of a street. Only participants who reported no discomfort or motion sickness after this test proceeded to the main experiment. Following this initial test, the EEG system was set up (see section “EEG preprocessing” for details), which took most of the time. Finally, the main experiment began with the eye-tracker's calibration and subsequent five-point validation.

The experimental duration lasted ∼40 min. The participants had 30 min to *ad libitum* explore the central city square (Fig. 1*B*, beige tiles) under the instruction to behave naturally as if waiting for a friend. Every 5 min, the exploration was paused for eye-tracker validation and recalibration and for the participant to take a break if needed. After each pause, participants were returned to their previous location in the city.

### The virtual environment

The virtual environment was modeled to resemble a city center, populated with various background objects (e.g., buildings, foliage; Fig. 1*B*) and 140 pedestrians (Fig. 1*C*). Pedestrians were sourced from the Adobe Mixamo collection (Mixamo, 2008), displaying varied activity and animation levels ranging from stationary and static to actively moving throughout the city. The pedestrians were designed to represent typical behaviors such as shopping, meeting friends, or relaxing on benches. The pedestrians did not react to the participants other than avoiding movement collisions. The active-moving pedestrians moved along predefined paths. Each object in the virtual environment had a collider, an invisible box or sphere marking the outline of an object, attached to them, with pedestrians having a separate collider for their heads and the rest of their bodies. This allowed us to separately investigate the neural response toward the heads and bodies of the virtual avatars. Participant movements within the virtual environment were programmed to mimic real-life displacements controlled by the participants’ head orientation and matched in speed to the moving pedestrians. The dimensions of the virtual city matched the real world, with one unity unit corresponding to 1 m, allowing us to indicate distances using meters.

### Using gaze events to determine EEG trial onsets

**Figure 2.**
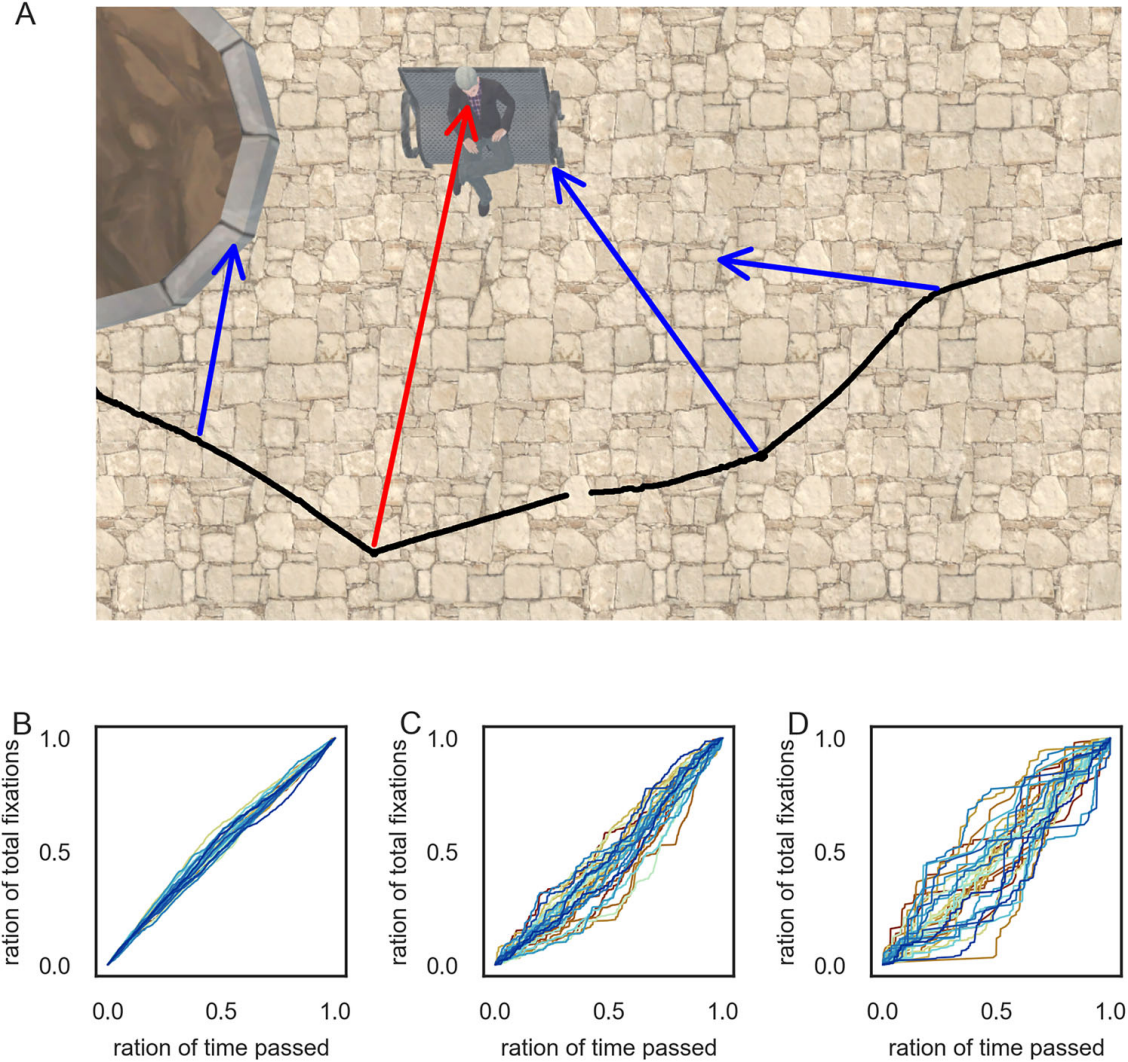
Distribution of gaze events. ***A***, An example of different fixations on different objects is plotted on top of the corresponding image of the city center. As a note, the data were slightly adjusted for visualization purposes only. The black line corresponds to the participant's movement path, and the arrows correspond to a few selected fixations during this duration. A fixation on the face of a pedestrian is highlighted in red. The blue arrows correspond to fixations directed at different background objects. ***B–D***, The distribution of background (***B***), body (***C***), and head (***D***) fixations over time, displayed as cumulative distribution functions. Each line corresponds to one participant.

We recorded EEG and eye-tracking simultaneously to use the timing of gaze events (fixations or saccades) as trial markers. Due to the absence of external stimulus onsets (or comparable events), we consider the data recorded during fixation (or saccade) and its immediate temporal context as a “trial.” This allowed us, for example, to investigate fixation ERPs (Dimigen, 2020; Gert et al., 2022). To this end, accurate detection of event onsets (fixations and saccades) in the eye-tracking data was essential. Therefore, we employed a velocity-based eye–tracking algorithm for free-viewing and free-exploration in a virtual environment, which corrects translational movement information superimposed on the eye movement data (Nolte et al., 2024; based on Voloh et al., 2020; Dar et al., 2021; Keshava et al., 2023). Applying this algorithm allowed us to differentiate between gazes (eye-stabilization movements, from now on, simply referred to as fixations) and saccades. In detail, the continuous eye-tracking data were segmented into smaller intervals (Dar et al., 2021), and a data-driven threshold was calculated for each of these intervals (Voloh et al., 2020; Keshava et al., 2023). Consecutive samples exceeding this threshold were classified as a saccade, and samples below the threshold were classified as fixations. This process resulted in a sequential identification of saccades and fixations throughout the entire recording. These events could then be used as trial onsets for the EEG analysis. Specifically, we compared ERPs aligned to fixation and to saccade onset, where the latter used the saccade onset preceding each fixation as the trial onset (Amme et al., 2024). This approach allowed us to compare identical trials, differing by a time shift: the time point zero in saccade-onset trials happened several milliseconds before the corresponding time point of fixation-onset trials. Consequently, if events, such as small saccades and the matching subsequent fixation onsets, were not detected, they would be present in and affect both types of ERPs similarly. The trials for both fixation- and saccade-onset ERPs were split into three distinct stimulus categories: heads, corresponding to fixations on the heads of pedestrians, bodies, and background stimuli, encompassing everything that was not a pedestrian, allowing us to investigate the presence of an N170 effect in a free-viewing experiment conducted in VR. For saccade-onset ERPs, we used the stimulus category of the fixation directly succeeding the saccades. A sequence of a participant's walking path and a few selected fixations can be seen in Figure 2*A*.

### Temporal alignment of EEG and eye-tracking data

Aligning the EEG and eye-tracking data worked; however, visual inspection indicated a small constant linear drift between the overall EEG and eye-tracking (unity) timelines. To correct this drift, we calculated the difference between the first EEG and eye-tracking timestamps and between the last ones, computed the deviation between these differences, and applied it linearly to the eye-tracking timeline (Nolte et al., 2024). Twenty-one subjects displayed a more substantial drift, requiring the start–end deviation up to four times or to adjust the timeline by one (11 ms) or two (22 ms) sample(s), according to the 90 Hz sampling rate, over the course of a 30 min experimental session. Notably, this drift correction was identical for fixation and saccade onsets. The final dataset only included subjects for which we were confident in aligning the two data streams (also see above, Subjects).

### EEG preprocessing

Preprocessing was performed in MATLAB (R2024a) using the EEGLab software (Delorme and Makeig, 2004; version 2020.0). EEG data were first loaded into MATLAB, channels were renamed according to the 10-5 BESA standard system, and empty channels were removed. We then imported a separate trigger file containing all relevant fixation or saccade-onset events derived from our eye-tracking data (see above, Using gaze events to determine EEG trial onsets, for a detailed explanation). We applied a low-pass filter at 128 Hz and a high-pass filter at 0.5 Hz (pop_eegfiltnew, using a hamming window; Widmann et al., 2015). Following the recommendation of Klug and Kloosterman (2022), we downsampled the EEG data from 1,024 to 500 Hz to apply a line noise filter from the “zapline plus” plugin (Klug and Kloosterman, 2022, based on de Cheveigné, 2020). We conducted this procedure to automatically remove spectral peaks ∼50 Hz and, separately, 90 Hz. Then, ensuring the data were referenced to the average reference, we applied automated cleaning of noisy channels and data segments using the “clean_rawdata” plugin (Kothe et al., 2019). Our data included active movement and contained more noise than expected in a classic stationary laboratory setup. To this end, we chose to apply a conservative burst criterion of 20, referring to the standard deviation cutoff for the removal of bursts via artifact subspace reconstruction. Removed noisy segments were saved to be used by the unfold toolbox (Ehinger and Dimigen, 2019; for a detailed description, see below, EEG analysis). After channel removal, the clean dataset was rereferenced to the average reference once more. Using the AMICA plugin (version 15, Palmer et al., 2012), we performed an independent component analysis (ICA) on the cleaned data to identify and remove muscle, eye, heart, or remaining line or channel noise. For this step only, we high-pass filtered our data to 2 Hz (Dimigen, 2020). Components labeled with 80% muscle activity or above (mean, 16 components; SD, 7.407) or >90% of other noise (ocular movement, mean, 2.121; SD, 0.331; channel noise, mean, 0.303; SD, 0.529; cardiac artifact, mean, 0.060; SD, 0.242; line noise, mean, 0.030; SD, 0.174), as identified by ICLabel (Pion-Tonachini et al., 2019), were removed automatically. ICA weights were then transferred to the dataset filtered at 0.5 Hz. Finally, we interpolated the missing channels (spherical interpolation). The described procedure was repeated for all subjects before we applied further statistical analysis.

### EEG analysis

First, we analyzed and compared ERPs aligned to fixation and saccade onsets, investigating the ERP waveforms for −300 to 500 ms surrounding each event for individual subjects and the averaged ERPs across subjects.

Next, to account for and correct the effect of overlapping events due to our free-viewing paradigm, we used a linear model implemented by the unfold toolbox (Ehinger and Dimigen, 2019) with the current event factor and the levels of background, body, and head. This overlap correction was applied for −500 up to 1,000 ms surrounding saccade onsets (Gert et al., 2022). As we investigate differences in saccade-onset ERPs, we did not model saccade amplitudes due to their high correlation with saccade durations (Harris and Wolpert, 2006; Guadron et al., 2022).

To investigate differences across categories at all electrodes and time points (−500 to 1,000 ms surrounding saccade onset), we conducted a one-factor repeated–measure ANOVA (1 × 3: head, body, and background), with an alpha level set at 0.05. To account for the multiple-comparison problem, we applied a cluster-based permutation test incorporating threshold-free cluster enhancement (TFCE), as implemented via the ept_TFCE MATLAB toolbox (Mensen and Khatami, 2013). We performed 10,000 permutations, randomizing data across the three factors for each permutation, followed by a one-factor repeated–measure ANOVA. The resulting *F* values were enhanced using TFCE (parameters *E* = 0.666; *H* = 1) based on recommendations for *F* statistics (Mensen and Khatami, 2013). This process generated an empirical null distribution (*H*0) of TFCE-enhanced *F* values, and the maximum *F* value across channels and time points for each permutation was recorded. The observed TFCE-enhanced *F* values were then compared with the empirical distribution, with statistical significance determined as values exceeding the 95th percentile of the null distribution.

### Assessment of face stimuli characteristics

To validate our stimuli, we conducted an online survey with a separate group of 12 participants (eight females, zero diverse; mean age, 31.62 ± 13.30 years). A total of 40 randomly selected facial images were shown: 10 of our avatars and 30 images selected from Gorlini et al. (2023), consisting of 10 each from three categories: unrealistic, semirealistic, and realistic faces. Participants rated each image on three indices using a validated questionnaire developed by Ho and MacDorman (2010): humanness (six items), eeriness (eight items), and attractiveness (four items), with semantic differential items assessed using a five-point Likert scale. For all stimulus categories, we calculated average scores for each participant for each index. Statistical differences were evaluated using a separate Friedman test per index.

### Code accessibility

The code described in the paper is freely available online at https://github.com/debnolte/saccade-onset_ERPs_of-face_perception_free-viewing_VR. The code is available as Extended Data.

10.1523/ENEURO.0573-24.2025.d1 Data 1 The code used for the creation of this manuscript. Download Data 1, ZIP file.

## Results

### Gaze events

Before analyzing ERPs, we first compared the different gaze events by examining the median and median absolute deviation (MAD) across various aspects of each category. One clear difference between the three categories—background stimuli, bodies, and heads—was the number of trials. Background stimuli had the most trials, with a median of 3,968 ± 492. Notably, the body and head categories had ∼10 times fewer trials than the background category, with bodies averaging 741 ± 221 trials and heads 151 ± 133 trials. Although there was considerable between-subject variation in the number of trials for both the body (min, 290; max, 1,296) and head (min, 17; max, 913) categories, the number of fixations directed at bodies and heads was not significantly correlated across participants (*r* = 0.184; *p* = 0.305). This indicated that it was not simply that some subjects gazed at pedestrians overall more or less; instead, some subjects focused more on heads, while others directed more fixations toward bodies. Despite the differences in trial counts, fixations in all three categories were equally distributed across the entire experimental duration (see cumulative distribution functions, Fig. 2*B*–*D*). This balanced distribution was essential, enabling comparison across the three categories without adjusting for differences in experimental duration or participant fatigue. When examining the median and MAD for event durations, fixation durations were similar across all three categories: background stimuli (0.186 s ± 0.013), bodies (0.2 s ± 0.016), and heads (0.178 s ± 0.023). In contrast, saccade durations differed, with background stimuli having the largest saccade durations (0.076 s ± 0.003), followed by bodies (0.066 s ± 0.001) and heads with the shortest saccade durations (0.056 s ± 0.015). A similar pattern emerged for saccade amplitudes, where saccades toward background stimuli had the highest amplitudes (12.119° ± 2.104), followed by bodies (7.212° ± 1.618) and heads (5.35° ± 1.491). These findings highlighted that, despite similarities in fixation durations and their temporal distribution, the different stimulus categories were associated with different saccade patterns and varying numbers of events.

**Figure 3.**
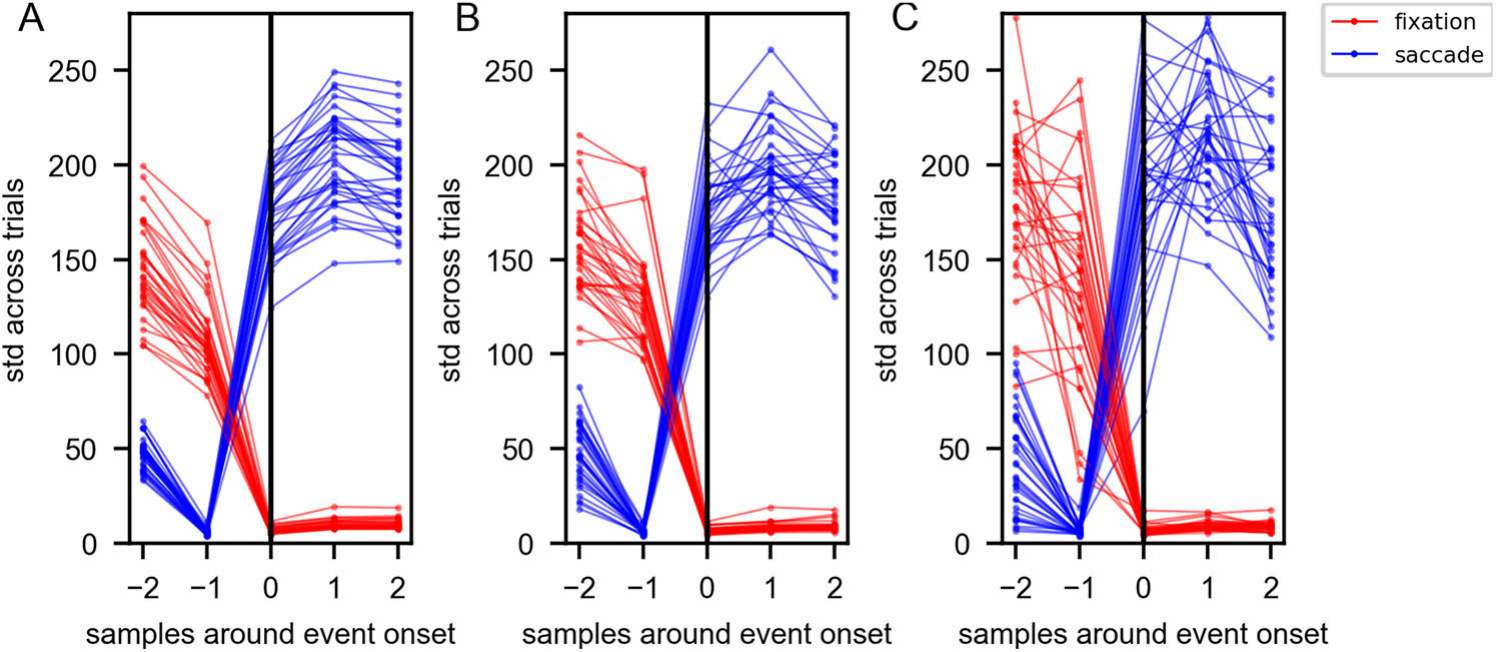
Variation of velocities around event onsets. The deviation of angular velocities surrounding event onsets is displayed for (***A***) background, (***B***) body, and (***C***) head trials. The *x*-axis displays samples around event onsets, with the fixation and saccade onsets aligned and marked by a black line. Velocities aligned to fixation onsets are shown in red, while those aligned to saccade onsets are displayed in blue. Each line corresponds to one participant, displaying the standard deviation across all trials.

Next, to investigate whether fixation and saccade onsets are subject to a bias inherent in the eye movement classification algorithm, we analyzed the distribution of eye movement velocities at event onsets (Fig. 3). Specifically, we examined the variability across trials. If we assume that event onsets are well defined, we would expect to observe a low variability of velocities around these onsets. In contrast, if the event onsets are not clearly defined, we would anticipate a higher variability. By comparing the variability of velocities at fixation and saccade onsets, we aimed to determine whether our classification algorithm was more precise in defining one type of event over the other. As shown in Figure 3, the velocities at fixation onsets exhibit a relatively low variability within and also across subjects. The velocities at saccade onsets display a similar but time-shifted distribution, with low variability within and across subjects, one sample before the saccade onset followed by a high variability at the saccade onset. Furthermore, the distributions of data for either event do not overlap. These observations provide support that the classification of fixations and saccades is not influenced by an obvious bias.

### Comparing fixation- and saccade-onset ERPs

**Figure 4.**
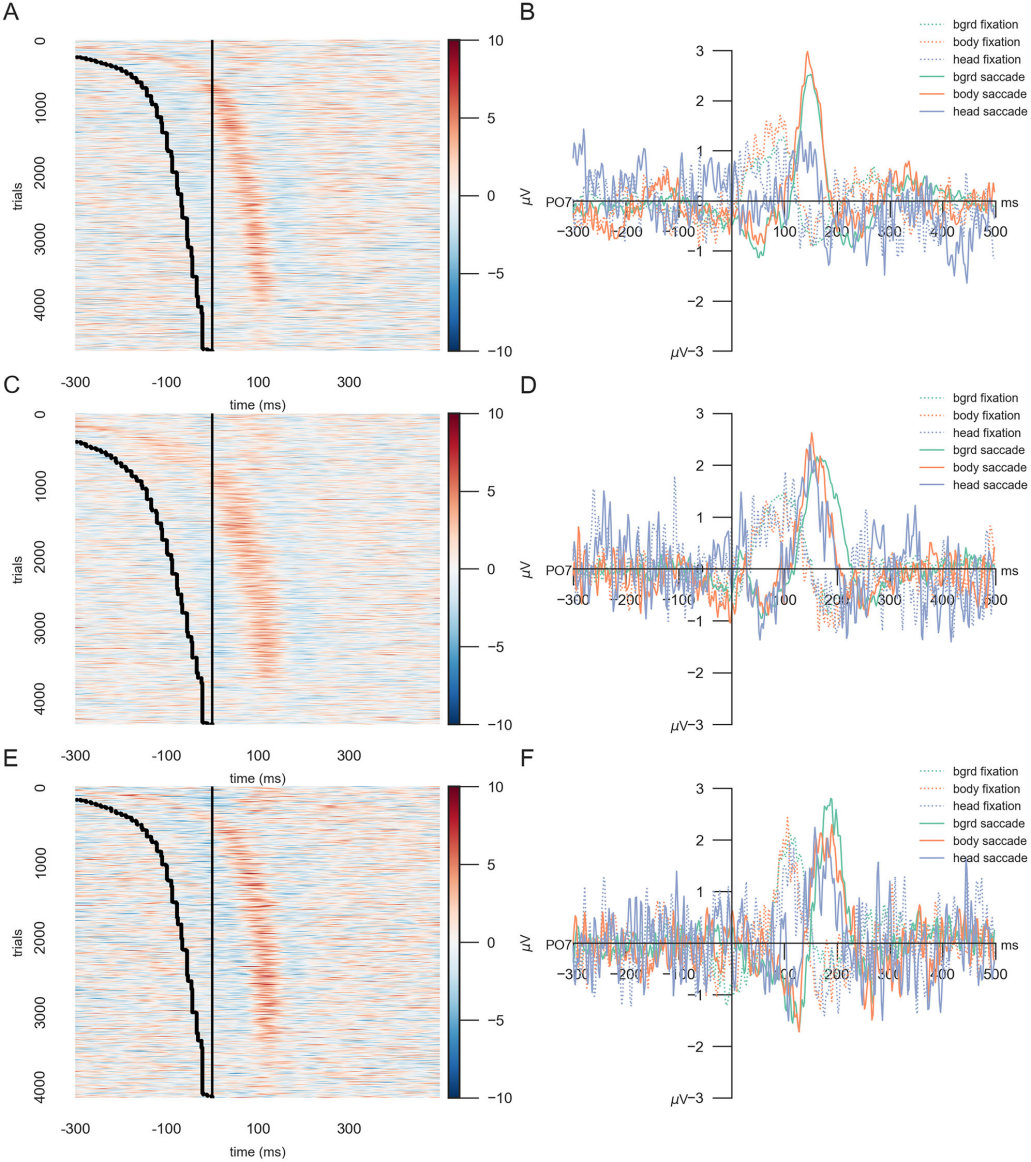
Fixation- and saccade-onset ERPs for a single subject. ***A***, All trials of one subject at electrode PO7, aligned to fixation onset, are sorted according to saccade duration, with the first trials (top of the *y*-axis) having the longest saccade durations. Red indicates positive, and blue indicates negative amplitudes. The trials are plotted over time. The black vertical line corresponds to the fixation and, therefore, trial onset. The trials are smoothed for visualization with a Gaussian filter of 2 in the *y* direction and a Gaussian filter of 5 in the *x* direction. The black-dotted line represents the saccade onset in each trial. ***B***, Fixation- (dotted lines) and saccade-onset (solid lines) ERPs of the same subject for electrode PO7. The different categories, background, body, and head, are indicated by the different colors. ***C***, ***D***, The same plots for a second and (***E***, ***F***) for a third subject.

In investigating ERPs, we first examined single subjects to compare the difference between fixation and saccade-onset ERPs, following the approach of Amme et al. (2024). For visualization purposes, we selected three representative subjects (Fig. 4). The first subject (Fig. 4*A*,*B*) had 4,620 trials, split into 3,779 background, 747 body, and 194 head trials. The second subject (Fig. 4*C*,*D*) had 3,802 background, 609 body, and 151 head trials, while the third subject (Fig. 4*E,F*) had 3,797 background, 792 body, and 361 head trials. To compare the difference between fixation- and saccade-onset ERPs, we sorted each subject's fixation-onset trials by the duration of the preceding saccade, in line with Amme et al. (2024). The EEG data were aligned and epoched using fixation onsets and then ordered based on saccade durations. Figure 4, *A*,*C*, and *E*, shows the results: fixations onsets are marked by the straight black lines at zero, while saccade onsets are indicated as the preceding curved black lines. If fixation onsets were the optimal alignment points, we would expect the P100 amplitude peaks to form a straight line 100 ms after the fixation onset. However, across all three subjects, the P100 amplitude peaks followed the curved saccade-onset trajectory, suggesting that saccade onsets provide more suitable time points for aligning individual trials in our free-viewing experiment. Interestingly, trials with very short saccades visually differ from longer saccades, potentially due to smaller changes of the visual input, smoothing, or an overlap of saccadic and fixation activity. The notion that saccade-onset ERPs were more temporally precise was further supported by time-shifted but higher P100 amplitudes compared with smaller, more smeared-out fixation–onset ERPs (Fig. 4*B*,*D*,*F*). Notably, the saccade-onset ERP waveform of the head category had higher noise levels than the background and body categories. Overall, the single-subject results supported the idea that saccade-onset ERPs might be a better-suited analysis method than fixation-onset ERPs for this type of experiment.

**Figure 5.**
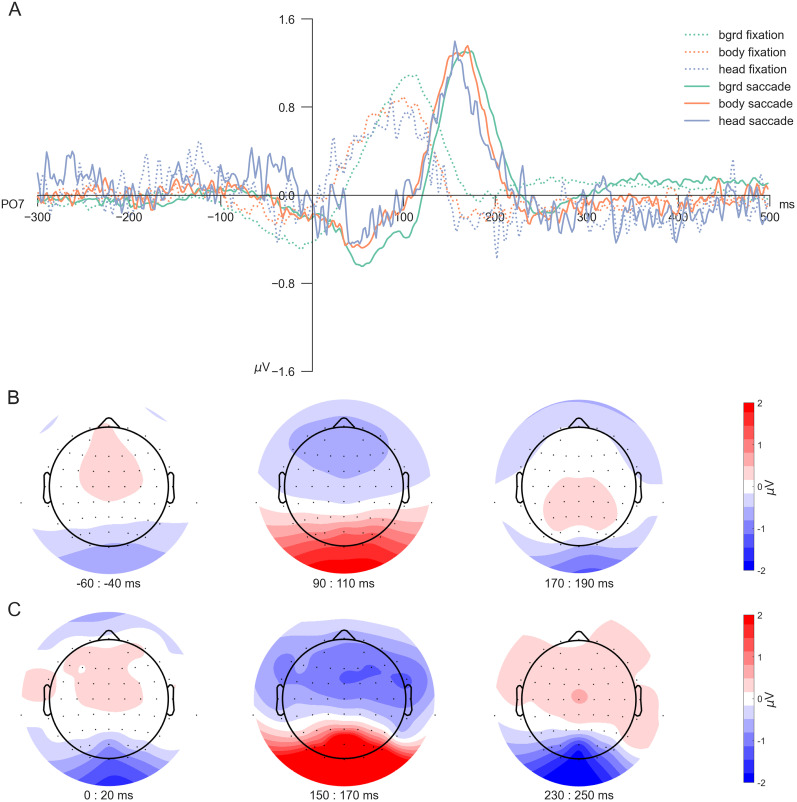
Fixation- versus saccade-onset ERPs. ***A***, Across-subject ERPs at channel PO7 for all three categories for the two different onsets: fixation-onset ERPs shown with dotted lines, saccade-onset ERPs with solid ones. ***B***, ***C***, Topoplots across all trials irrespective of the stimulus category (the average of all background, body, and head trials) of (***B***) fixation-onset and (***C***) saccade-onset ERPs, shown for three distinct time points: before the P100, around the P100, and at the N170. For visualization purposes, we selected time intervals based on the visual inspection of fixation- and saccade-onset ERPs, maintaining a constant difference between fixation and saccade onsets.

Next, we investigated the difference between fixation- and saccade-onset ERPs across subjects. For this, we first averaged within subjects to account for the high variability of head trials and then averaged across subjects. Fixation-onset ERPs (Fig. 5*A*, dotted lines) show a broad P100 component across all three stimulus categories, with background stimuli evoking the highest P100 peak and head stimuli the lowest. In comparison, saccade-onset ERPs (Fig. 5*A*, solid lines) are shifted in time but exhibit higher amplitudes across all stimulus categories and P100 peaks that are more temporally focused. Interestingly, the differences in P100 peaks between the stimulus categories seen in fixation-onset ERPs disappear with saccade-onset alignment. Like the single-subject results, the head category appears to be the noisiest, regardless of the alignment. The topographical analysis across all channels (Fig. 5*B*,*C*) supported this observation, highlighting that the preference for saccade-onset ERPs is not restricted to only the single, selected electrode. ERPs aligned to the saccade onset elicit higher amplitudes across occipital electrodes than those aligned to the fixation onset. These findings support the usage of saccade-onset versus fixation-onset alignment in EEG analyses due to their impact on the overall ERP curve. Saccade onsets lead to more well-defined ERPs, especially concerning the P100 component.

To statistically compare fixation- and saccade-onset ERPs, we followed the approach of Amme et al. (2024), grouping the data into 10 equally sized bins based on saccade duration. Within each bin, ERPs were averaged first within participants and then across participants. We identified the half-maximum point of the P100 slope for each binned ERP waveform. We then calculated the standard deviation of the half-maximum time points across bins, separately for fixation- and saccade-aligned conditions. The standard deviation was 19.82 ms for fixation-onset ERPs and 8.32 ms for saccade-onset ERPs. A paired-sample *t* test confirmed that this difference was statistically significant (*t*_(9)_ = 7.828; *p* < 0.0001), indicating that saccade-onset alignment yields a more temporally stable estimate of the P100 component.

### Comparing head, body, and background trials of saccade-onset ERPs

**Figure 6.**
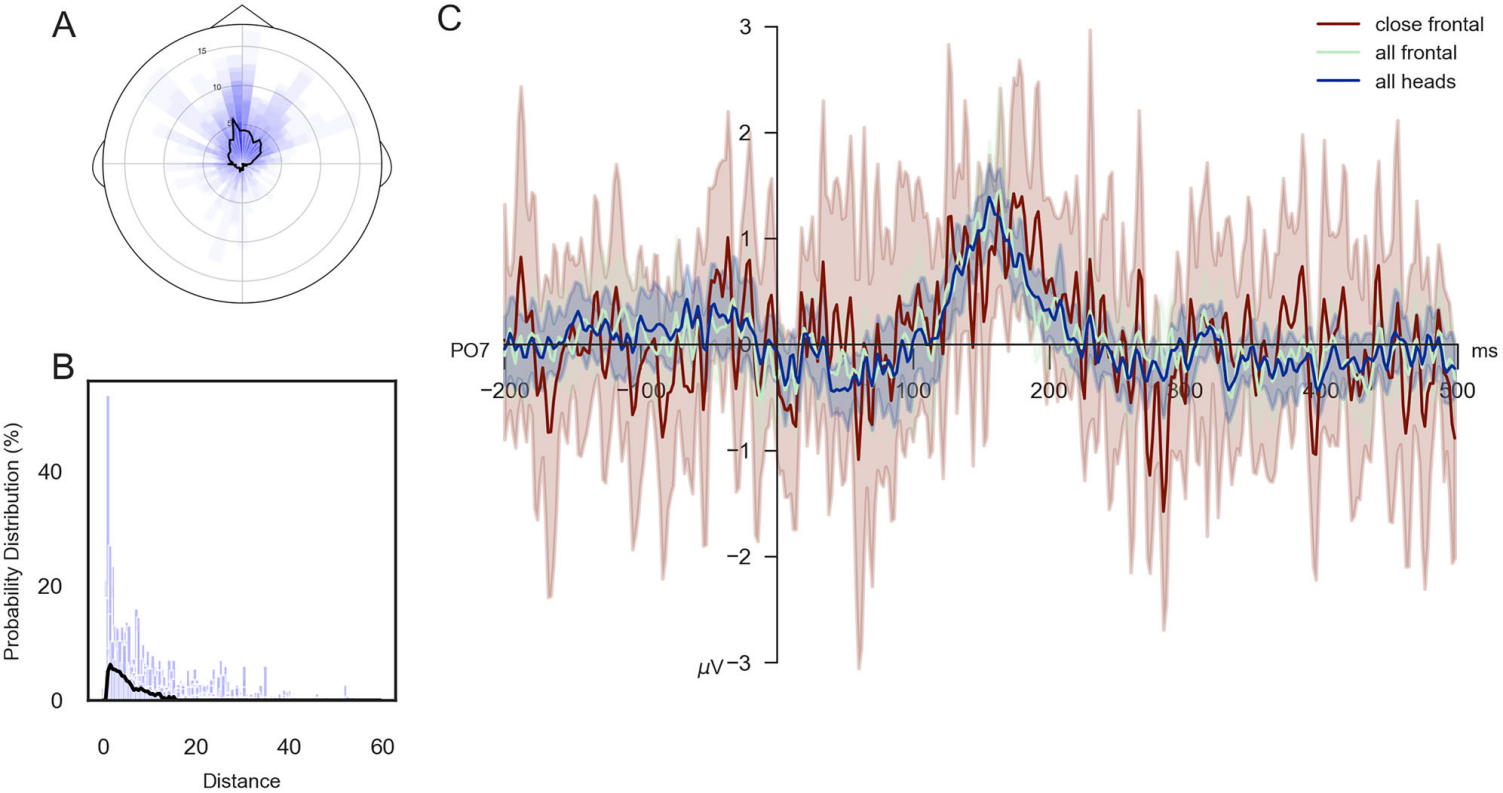
Rotation and distance effects on head saccade-onset ERPs. ***A***, The distribution of viewed orientations of pedestrians’ heads is displayed as percentages; the top of the plot corresponds to face fixations, while the bottom refers to fixations on the back of the head. The distribution of the individual participants is displayed by blue bars, and the black line indicates the median distribution across participants. ***B***, The distribution of distances is shown for each participant in blue bars, and the black line represents the median distance across participants. ***C***, The ERP for all head fixations is displayed in blue. Frontal head fixations, corresponding to those within 90° of the front of the head, are shown in green. Frontal fixations on pedestrians at a distance of 5 m or less are depicted in red. Each line and the corresponding confidence intervals represent the average across participants.

Before investigating the presence of an N170 effect, typically associated with the perception of faces (Rossion and Jacques, 2008; Eimer, 2011), we assessed that aggregating all fixations on heads, irrespective of viewing angles, does not obscure any potential differences. To this end, inspecting the viewing angle distribution across participants (Fig. 6*A*) revealed that most fixations are directed toward pedestrians’ faces. Specifically, computing the circular mean within participants, followed by the circular mean and the circular standard deviation across participants, resulted in average viewing angles of 13.210 ± 30.868°. Additionally, to exclude viewing distance as a potential influence, we computed the median and MAD across participants (5.209 ± 2.591 m; Fig. 6*B*), indicating that most heads are viewed at a close range. When we investigated the saccade-onset ERPs of fixations on heads (151 ± 133 trials) compared with those on only faces (72 ± 64 trials), no visible differences other than a slight increase in noise levels emerged, suggesting that aggregating all head fixations is valid. Only inspecting frontal faces viewed at a close distance (<5 m) resulted in fewer trials (39 ± 38 trials) and an ERP with high noise levels, making the ERP curve challenging to interpret. Overall, our results indicate no discernible differences elicited by viewing angles of distances, confirming that aggregating head fixations is appropriate.

**Figure 7.**
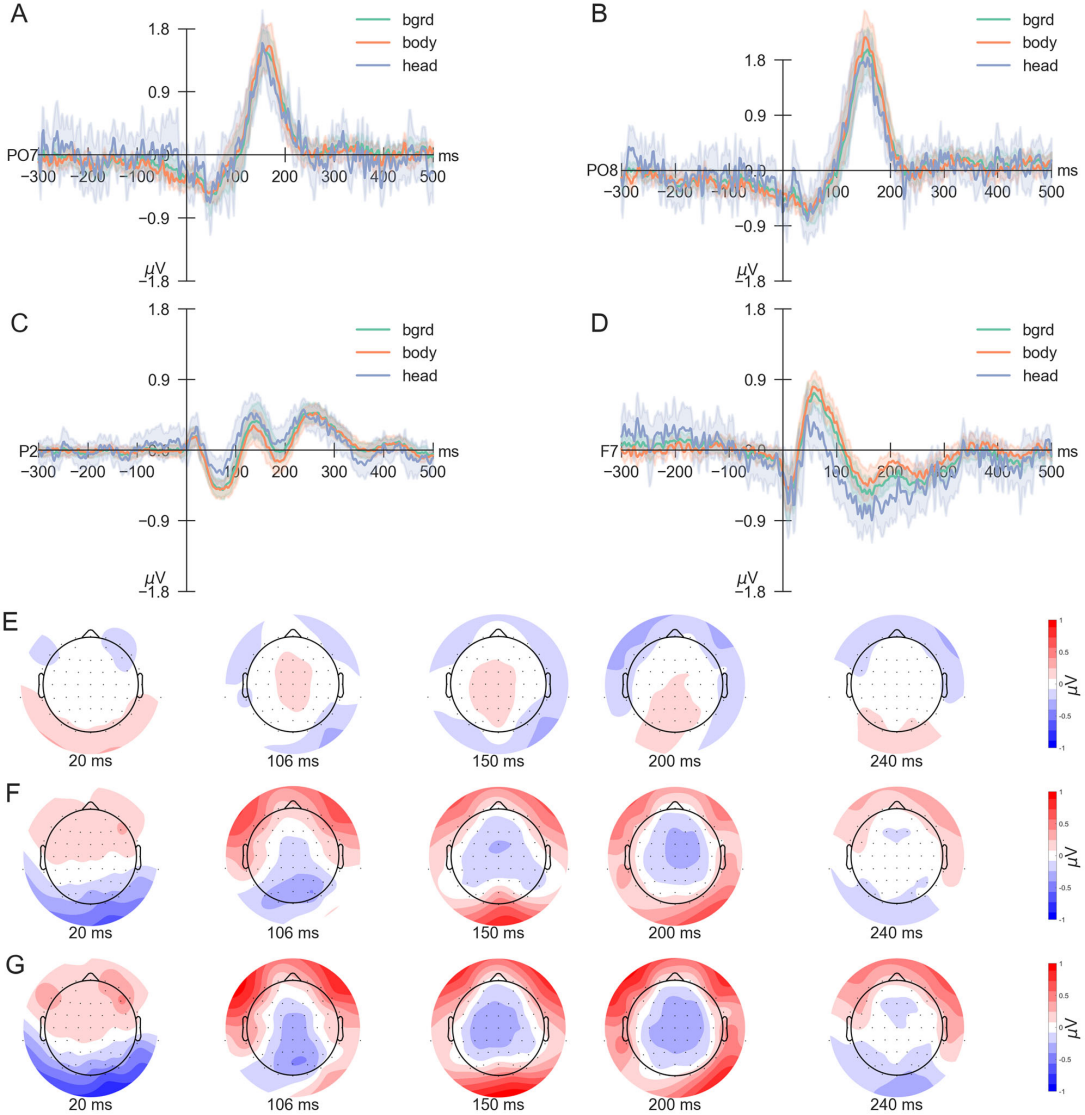
Saccade-onset ERPs for all three categories. ***A–D***, The across-subject average saccade-onset and deconvoluted ERPs were separated for background, body, and head trials. The average ERPs and corresponding confidence intervals indicating the average across participants are shown for four different electrodes: PO7 (***A***), PO8 (***B***), P2 (***C***), and F7 (***D***). The stimulus categories are indicated by different colors. ***E–G***, Difference plots of two categories at distinct time points after saccade onset: 20, 106, 150, 200, and 240 ms. The topographic plots are shown for the average difference between (***E***) background minus body, (***F***) background minus head, and (***G***) body minus head trials.

To examine category-specific differences of saccade-onset ERPs, we first focused on channels discussed in previous literature (Gert et al., 2022), particularly PO7 (Fig. 7*A*) and PO8 (Fig. 7*B*). Comparing the deconvoluted potentials across categories at these channels revealed no visible discrepancies, with only minimal, if any, negative deflection following the initial positive peak, a time-shifted P100. In contrast, other electrodes displayed notable differences between categories. For instance, at electrode P2 (Fig. 7*C*), the head trace diverged from the body and background traces after the saccade onset until reaching peak amplitudes ∼150 ms. Similarly, at frontal sites such as F7 (Fig. 7*D*), distinctions between the head compared with both background and body categories were visible around the fixation onset, ∼50–80 ms after the saccade onset. Notably, across all four electrodes, the head category exhibited a higher noise level and more considerable between-subject variability than the other two categories, most likely caused by the lower number of trials. This variability of the head category and the observed topographical distinctions required a statistical approach beyond traditional measures such as peak-to-peak comparisons.

**Figure 8.**
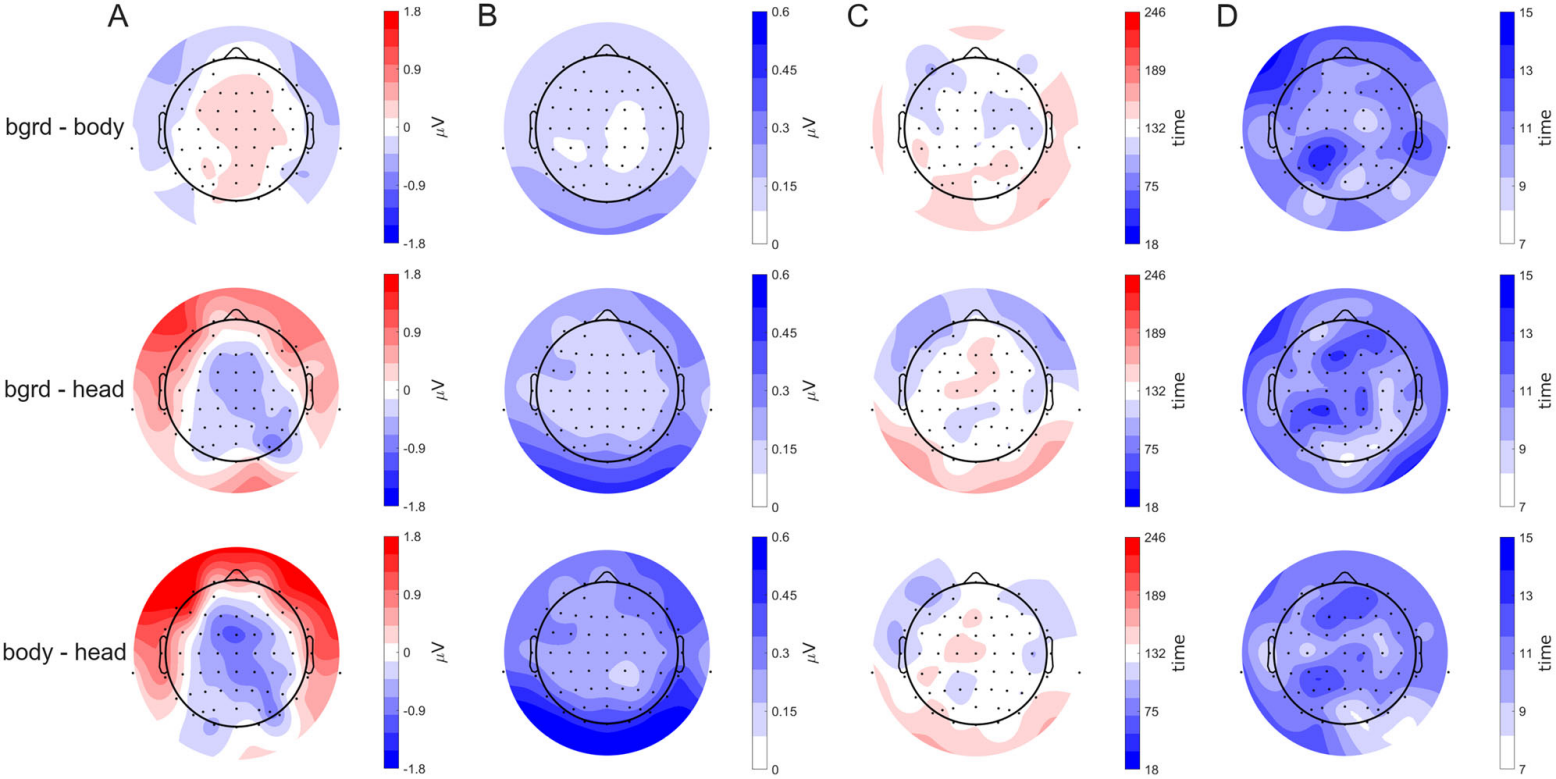
Measures of the effect size of saccade-onset ERPs. For each of the three differences (background–body, background–head, body–head), the results of the TFCE are displayed. ***A***, The maximum contribution averaged over participants is displayed for each electrode. The results are given in microvolt. ***B***, The corresponding standard errors of the mean for each maximum distribution are displayed. ***C***, The time points for the maximum contributions of each electrode are shown. The color bar corresponds to the time interval of the significant cluster, lasting from 18 to 246 ms. ***D***, The corresponding standard error of the mean for the time of each maximum contribution is displayed. In addition, smaller clusters can be found in the Extended Data Figure 8-1.

Accordingly, we employed a mass univariate analysis to examine category-specific differences across electrodes and time. Specifically, testing for significant differences using TFCE (correction for multiple comparisons with *α* < 0.05) revealed a significant cluster most compatible with an effect spanning from 18 to 246 ms and encompassing all electrodes. The cluster started at channel F7 at 106 ms (see Fig. 7*D* and the second difference plot in Fig. 7*E*–*G*) shortly after fixation onsets, with a median difference between background and body of −0.174 μV, a median difference between background and head of 0.505 μV, and between body and head of 0.658 μV at this time point and electrode. Of note, while this cluster effect starts quite early, it does not reach negative times. Inspecting temporal differences between categories further highlighted this effect (Fig. 7*E–G*), revealing a gradual shift in differences between categories, primarily driven by the head category (Fig. 8*A*). Notably, while the maximum contribution is primarily due to differences between the head and either of the other two categories (Fig. 8*A*,*B*), the timing of each maximum contribution differs little for all three contrasts. Specifically, it can be observed that frontal electrodes have their highest contribution on average around the saccade onset, while occipital electrodes have their highest contribution slightly later (Fig. 8*C*,*D*). Additional smaller clusters were found. As we wish to avoid a multiple-comparison problem, we do not report this information here but include it as Extended Data Figure 8-1. Overall, these findings support the presence of category-based differences throughout the entire temporal window and all electrode sites, with the highest differences visible around the saccade onset.

10.1523/ENEURO.0573-24.2025.f8-1 Figure 8-1 Description of additional clusters found using TFCE. Download Figure 8-1, DOCX file.

### Assessment of face stimuli characteristics

**Figure 9.**
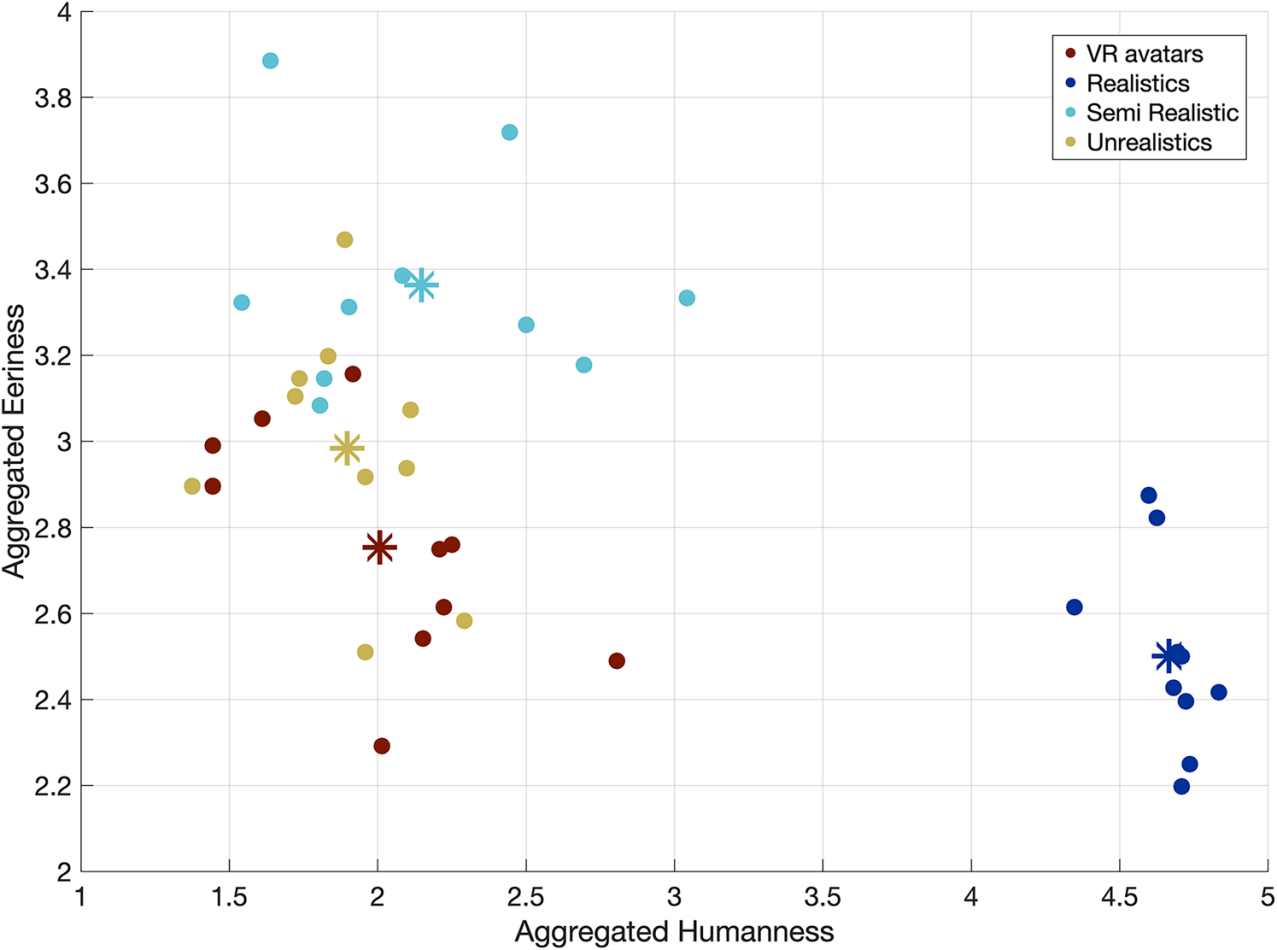
Assessing the uncanny valley effect. For each image (10 per category—40 in total), we computed the average score of eeriness and humanness across participants. The four categories (VR avatars, unrealistic, semirealistic, and realistic) are displayed in different colors. The mean of each category is indicated by a star. The statistical results can be found in Extended Data Figures 9-1 and 9-2.

To assess whether the visual appearance of our virtual avatars may have contributed to reduced face fixations, we conducted a supplementary face rating study. A Friedman test confirmed significant differences between stimulus categories for humanness (*χ*^2^_(3)_ = 24.3; *p* < 0.001), eeriness (*χ*^2^_(3)_ = 15.4; *p* = 0.001), and attractiveness (*χ*^2^_(3)_ = 27.5; *p* < 0.001; see Fig. 9 and Extended Data Figs. 9-1, 9-2 for details). Post hoc tests revealed differences between our avatars and the unrealistic category in terms of attractiveness; they aligned most closely with the unrealistic category in terms of humanness and eeriness. These findings suggest that our avatars do not strongly induce an uncanny valley effect.

10.1523/ENEURO.0573-24.2025.f9-1 Figure 9-1 Average ratings of the different picture categories on three indices. Download Figure 9-1, DOCX file.

10.1523/ENEURO.0573-24.2025.f9-2 Figure 9-2 Results of the Friedman tests and post-hoc analysis for the three indices. Download Figure 9-2, DOCX file.

## Discussion

This study explored methodological considerations for using VR and EEG to investigate ERPs in a free-viewing task. We designed a three-dimensional virtual city populated with virtual pedestrians, allowing participants to navigate and visually explore as they would in a real-world setting. Our findings align with a recent MEG study (Amme et al., 2024) showing that saccade-onset locked ERPs offered more consistent and interpretable results than traditional fixation-onset ERPs, particularly during time windows associated with P100 responses. This finding suggests that saccade onsets serve as more physiologically meaningful triggers for understanding visual processing, marking the initiation of critical neural mechanisms that shape ERP responses (Katz et al., 2020; Amme et al., 2024; Gordon et al., 2024). The underlying processes may be influenced by preparatory activity or predictive planning (Crapse and Sommer, 2008; Wurtz et al., 2011; Katz et al., 2020) or may be guided by attentional shifts (Hoffman and Subramaniam, 1995; Kowler et al., 1995; Deubel and Schneider, 1996; Rolfs et al., 2011), which are linked to enhanced visual discrimination (Deubel and Schneider, 1996). These mechanisms can be associated with the brain preparing for anticipated visual input before initiating the movement (Rao and Ballard, 1999; Friston, 2010), suggesting that early neural activity related to visual selection and saccade preparation might occur before the fixation onset (Henderson, 2017). In contrast, fixation-onset triggers might capture neural activity after the initial processing of visual input. As such, utilizing fixation onsets for self-initiated eye movements could lead to misinterpretations of visual processing timing and dynamics. These results support the critical role of self-initiated eye movements in priming the brain for incoming visual information (Amme et al., 2024; Gordon et al., 2024) and advocate for a methodological shift in free-viewing paradigms toward saccade-onset ERPs to improve our understanding of visual processing.

The selection of saccade onsets versus fixation onsets as triggers for ERPs carries significant methodological implications, particularly concerning the timing and interpretation of early components like the P100. Aligning ERPs to the saccade onset may result in a delayed P100 response compared with classical stimulus-locked ERPs, with the exact timing of this component in relation to saccade events requiring further investigation. Furthermore, the inherent variability in saccade characteristics, such as increased saccade amplitudes over time (Pannasch et al., 2008) or different saccade durations for different stimulus categories, could influence the analysis and interpretation of results. In contrast, later ERP components, dominated by lower-frequency dynamics, may be less affected by alignment but pose their own challenges for isolating saccade- versus fixation-related activity. Recent findings (Amme et al., 2024) show that saccade-onset ERPs contain residual fixation-locked activity, underscoring this complexity. We therefore do not argue against fixation-locked analyses in free-viewing designs but suggest that saccade-locked approaches provide greater temporal precision for early components and should be used alongside, rather than instead of, fixation-based alignment. Investigating whether the methodological shift will allow the comparison of neural responses to target categories associated with varying saccade durations is therefore essential.

To evaluate whether saccade-onset ERPs derived from VR eye movements would allow us to discern differences between various stimulus categories, we aimed to replicate the well-established N170 face effect (Rossion and Jacques, 2008; Eimer, 2011). Our results partially supported our hypothesis, showing a significant cluster of differences among the three experimental categories: heads, bodies, and background stimuli. These differences spanned much of the trial duration, including the P100 and N170 time windows across all electrodes, consistent with previous research (Gert et al., 2022), implying that neural processes associated with face processing (Rossion and Jacques, 2008; Freiwald et al., 2016; Gao et al., 2019) likely contribute to these differences. These results demonstrate the feasibility of using VR for examining category-based differences.

While our results indicate that combining EEG and eye-tracking in VR allows for studying differences in stimulus categories, several aspects must be examined to interpret our results. First, the observed face effect may be influenced by residual noise, particularly given that the cluster peak is located near the cluster's margin. While we aimed to minimize these factors through preprocessing, individual participant or trial variability may still contribute to the effect. Relatedly, we note that our decision not to model saccade amplitudes despite their known influence on the P100 effect may also be relevant in this context (Henderson et al., 2013; Ehinger and Dimigen, 2019; Guadron et al., 2022). Instead, we investigated differences in saccade-onset ERPs, aligning neural responses across categories while accounting for saccade durations, which strongly correlate with saccade amplitudes (Harris and Wolpert, 2006; Guadron et al., 2022). Given this correlation, we believe our approach did sufficiently control for saccade effects. However, further testing should rule out the saccade amplitudes’ contribution to the observed face effects. Here, a study contrasting categories with comparable amounts of trials would be essential.

In the current study, despite expecting interindividual differences in gaze behavior (Guy et al., 2019; Guy and Pertzov, 2023; Rubo et al., 2023), the high between-subject variability and a low number of head fixations for some subjects were surprising, given the changes in movement of the pedestrians stimuli, something known to capture attention (Abrams and Christ, 2003). The differences in trial counts and resulting varying noise levels made it impossible to test our hypothesis regarding the N170 effect directly. However, increasing the trial count might not have led to conclusive results, as visual inspection of our ERP traces revealed no observable negative peaks in occipital–temporal electrodes typically implicated in N170 research (Rossion and Jacques, 2008; Eimer, 2011).

Several factors may explain not observing a visible face-selective component in this study. First, many free-viewing studies show smaller or absent ERP components compared with stimulus-locked studies (Auerbach-Asch et al., 2020; Gert et al., 2022; Ladouce et al., 2022; Spiering and Dimigen, 2025), a difference that may be even more pronounced in VR-based research. As we did not account for previous fixation types, potential adaptation effects cannot be ruled out (Gert et al., 2022; Spiering and Dimigen, 2025). Future studies should therefore contrast saccade-onset ERPs to stimulus-locked ones to disentangle those effects (Amme et al., 2024; Spiering and Dimigen, 2025). Relatedly, overlapping (micro)saccade responses, undetectable with our eye-tracker's sampling rate, could temporally coincide with and mask the true N170 component, leading to its apparent absence (Spiering and Dimigen, 2025). Second, methodological aspects could play a role. While virtual faces have been shown to elicit an N170 effect, it is typically weaker than in two-dimensional studies (Kirasirova et al., 2025). As our stimulus set has not been systematically evaluated in a stimulus-locked experiment, future studies should investigate the presence of an N170 effect in such a setup using our or similar avatars. Beyond this, to our knowledge, this study is the first to examine face perception in a free-viewing, free-movement VR setup, introducing new variables such as attentional shifts or stimulus variability in a virtual environment, which may contribute to observing a nonvisible effect. Additionally, other methodological factors, such as the virtual environment or the density of visual stimuli, may contribute to the observed findings. Third, individual differences and hemispheric variations in the N170 response (De Vos et al., 2012) could introduce variability, rendering an across-subject investigation difficult. Finally, although fixation-locked ERPs to front-facing faces have previously revealed meaningful condition differences (Auerbach-Asch et al., 2020; Gert et al., 2022), we consider it unlikely that not observing a clear N170 effect in our data is due to the use of saccade-onset ERPs. Visual inspection of fixation-locked traces likewise did not reveal a distinct N170 component, and given the predominance of front-facing head stimuli, it seems unlikely that fixation-aligned analyses would have produced a markedly different pattern for rotational effects. Nonetheless, future studies explicitly designed to compare fixation- and saccade-onset ERPs (Amme et al., 2024) are needed to disentangle their respective contributions. Overall, determining the exact cause of an N170 face effect not being observable remains complex and warrants future investigation. These results highlight challenges when adapting lab-based methodologies to free-viewing studies and suggest the need for a methodological shift when moving toward experiments with higher ecological validity.

Several challenges have been associated with recording multimodal mobile data in a VR setting (Stangl et al., 2023). One of them, the alignment of various data streams, could be tackled by employing state-of-the-art amplifiers with a built-in connection or direct support for data synchronization tools such as LSL to improve the temporal accuracy of recordings further (Gramann, 2024). In this sense, effective and reliable synchronization would reduce alignment or drift issues that led to the exclusion of subjects’ data in the present study. Additionally, technological advancements could lead to the creation of even more realistic VR environments. With increasing visual realism and accuracy, undesired side effects of VR setups, such as motion sickness, could be reduced (Keshavarz et al., 2011; Kim et al., 2018; Clay et al., 2019; Rebenitsch and Owen, 2021). This process is likely to develop in the future. In contrast, increasing realism will also increase the possibility of an uncanny valley effect of virtual agents (Schindler et al., 2017), something future studies should carefully examine. In the current study, even though we have no indication that the faces of our avatars elicited an uncanny valley effect, we cannot exclude the possibility that virtual faces were avoided due to their appearance. Nonetheless, we recommend using artificial avatars, as they offer a high degree of interaction, modification (Schindler et al., 2017), or specific positioning (Sánchez Pacheco et al., 2025) compared with static images or video recordings. Further recommendations might be to record in smaller segments, allowing for higher control over the recorded data quality. Importantly, while facing challenges, setups combining VR, tracking, and brain imaging techniques offer great potential to provide novel insights into the interplay of action and perception by enabling dynamic, precise, and synchronous measurements across multiple domains and measurement devices (Baceviciute et al., 2022; Kim et al., 2023; Larsen et al., 2024). With these considerations in mind, we recommend using similar setups to address and re-evaluate research questions in settings with higher ecological validity (Parsons, 2015; Kothgassner and Felnhofer, 2020).

In conclusion, our results demonstrated the potential of VR-based, free-viewing paradigms combined with EEG to capture meaningful neural data, with saccade-onset–locked ERPs offering advantages over the fixation onset for ERP analysis. Employing saccade-onset ERPs, we observed significant differences between stimulus categories, providing a suitable approach for analyzing free-viewing data. However, variability in noise levels across categories and among subjects underscores the complex nature of free-viewing studies. While combining free-viewing in VR with EEG recordings is feasible and yields valuable data, significant methodological challenges remain. With this work, we highlight the need to develop further and refine measurement and analysis approaches to suit the needs of advanced experimental designs. Thereby, we aim to contribute to laying a foundation for future studies to advance the field of free-viewing or exploration paradigms in visual 3D environments further.

## Synthesis

Reviewing Editor: Frederike Beyer, Queen Mary University of London Decisions are customarily a result of the Reviewing Editor and the peer reviewers coming together and discussing their recommendations until a consensus is reached. When revisions are invited, a fact-based synthesis statement explaining their decision and outlining what is needed to prepare a revision will be listed below. The following reviewer(s) agreed to reveal their identity: Annika Ziereis. Note: If this manuscript was transferred from JNeurosci and a decision was made to accept the manuscript without peer review, a brief statement to this effect will instead be what is listed below.

As you can see from the detailed comments below, both reviewers find your manuscript timely and of interest to the field. Both also request more detail regarding some methodological aspects and presentation of results.

Reviewer 1

This manuscript aims to provide new insights into suitable methods for analyzing event-related potentials (ERPs) during ecologically valid task performance. Using VR goggles to control biometric signal measurements while creating realistic task scenarios is an intriguing approach. The study addresses a previously unexplored question of whether to compute ERPs based on fixation or saccade events, and has the potential to offer a practical methodology. However, realizing this potential requires providing more detailed explanations of the methodology.

While the manuscript flows well and is easy to read, the simplicity comes at the cost of sacrificing detailed descriptions of the methods. Providing additional methodological details is critical to ensuring reproducibility and scientific rigor.

Please note that this review reflects my perspective as a researcher with expertise in eye movement measurement and autonomic nervous system response acquisition in real-world scenarios but only a general knowledge of EEG.

Major Comments:

・The visualization for a single participant is a useful approach to outlining the overall narrative (Figures 3-A and B). However, it raises concerns about how this participant was selected, as it might significantly influence the impression of the overall results. Evidence supporting the claim that this participant represents a typical case, or a clear note that this visualization only serves illustrative purposes, should be added. This concern is especially pertinent as the participant in Figure 3C appears to be an outlier compared to others.

・The methods used to create Figure 3A require more detailed explanation. Although a prior study is cited (Amme et al., 2024), it would be ideal for the manuscript to stand alone in its methodological clarity. The cited study appears to be a preprint and not yet peer-reviewed, underscoring the need for the methods to be independently validated through this peer-review.

・While I acknowledge the difficulty of isolating factors in ecologically valid (noisy) experiments, additional clarification is needed. Is there a possibility that the results are explained by the algorithm that detects saccades and fixations?

Specifically:

・Could the detection algorithm's biases impact the results, such as favoring the detection of one type of event (saccade or fixation) over the other? The authors note elsewhere that fewer events lead to higher noise ratios when calculating ERPs. Could the differences in the number of detected events between conditions contribute to the observed effects?

・Some algorithms might not independently detect saccades and fixations, respectively, leading to equal numbers of events. However, even in this case, one type of event (e.g., saccades) might be more "pure," potentially explaining the observed differences in ERP alignment effectiveness. For instance, in such a biased case, fixation events might include fixation and small saccades, whereas saccade events may consist solely of clear saccades, favoring ERP alignment.

・Couldn't fixation-based alignment yield more precise ERP timing? Since saccades are not uniform in duration, could this variability affect the timing accuracy of ERP calculations?

・If saccade-based alignment improves timing precision, the authors should explain why this is the case.

Minor Comments

・The term "neuronal" is used in several places (e.g., page 2). As ERPs represent the sum of multiple neuronal activities, "neural" might be more appropriate. However, if native speakers find "neuronal" acceptable, this comment can be disregarded (I'm not a native English speaker).

・The manuscript could emphasize the advantage of VR goggles with integrated eye trackers in the methods section. While I know this, general readers might not understand which eye tracker was used, given the current description.

・In Figure 3B, "gaze" might be better replaced with "fixation" to avoid confusion.

・On page 8, there is a notation "(s." which appears to be a typographical error. Please correct this.

Reviewer 2

The present study investigated neural responses to heads and bodies of virtual agents compared to other visual objects in a free-viewing virtual reality environment. Here, the authors focused on advancing methodological considerations when facing typical challenges of investigating face processing in a naturalistic setup compared to controlled static setups. Although the initial objective of replicating the classic N170 face effect in the VR environment was not achieved, the results revealed significant category differences in the processing of heads, bodies, and other visual objects during the P100 and N170 time windows when using a cluster-based approach. The use of co-registered EEG and eye-tracking data allowed the authors to investigate the impact of saccade-onsets on ERP analysis. The results showed that ERPs based on saccade-onsets yielded clearer and more well-defined early brain responses compared to fixation onsets, consistent with recent findings from a MEG study by Amme et al. (2024).

The authors address a timely and relevant topic by investigating face processing in a more naturalistic setting, moving away from the traditional controlled laboratory environment. Their approach of using virtual reality (VR) to achieve better controllability and standardization is well-justified. Also, the authors have successfully presented their methodology and approach in a transparent and clear way, making the manuscript easy to read and to the point. The decision to preview the results at the beginning of the manuscript is unconventional, but in this case, it is effective in setting the reader's expectations, as the actual results diverge from the hypothesized outcomes. Finally, the authors demonstrate transparency by acknowledging potential limitations of the study which adds to the overall quality of the manuscript.

My main difficulties with the manuscript lie in the fact that it is not entirely clear whether the methodological aspect is the primary focus (saccade-onset vs. fixation-onset) or if the content is actually about face perception (or if it is only used as an application case). In either case, I feel a more detailed elaboration of the theoretical aspects and implications is missing:

a) In the first case, where the methodological aspect is the primary focus, I would have expected a discussion of the analytical approach, not only including one selected participant but the distribution of these patterns across the whole sample, discussion about the implications of using saccade-onset or fixation-onset as triggers for ERPs, particularly in relation to predictions (saccade planning), movement, and timing, and an investigation about the expected differences in processing windows compared to fixed stimulus presentations.

b) In the second case, where face perception is the primary focus, I would have liked to see the authors attempt to provide additional explanations for their results, particularly because the typical N170 face effect was not found. The authors themselves mention some possible reasons, but they do not explore or address these further to better understand the results:

1) It is mentioned that there may not be enough trials in which participants were looking at the head, and that there may have been a lot of variation due to the head orientation of the avatars and movement. While it may be inconvenient, it would be useful to know how often faces (seen relatively frontally from a rather close distance) were actually viewed. At least as a manipulation check, it would be good to see how the reaction to frontal faces was. If this is not possible, I wonder if this paradigm is suitable for the investigation of neural face processing (if faces were hardly viewed at all). Perhaps the controlled VR setup could be used for a different research question and a more specific focus on saccade-onset vs. fixation-onsets could be explored.

2) The effectiveness of the stimulus material is not addressed. Was this material previously validated (internally or externally)? Would the avatars be able to evoke the expected effects in a non-VR or static setup? Are the avatars perceived as aversive (uncanny valley), leading to avoidance of fixation? Perhaps a specific task for the participants could help ensure that they are viewing the faces, such as "find person X". Additionally, I wonder if the authors would advise in favor or against using artificial avatars (in comparison to video recordings of real faces) in future studies.

3) The authors do not provide a clear explanation for the findings, aside from the increased noise level. They cite a study by Gert et al. 2022, showing that a fixation-related N170 ERP has been found in previous free-viewing setups, including those with complex scenes. However, this is not the case in the current study. I would have expected the authors to provide a more detailed explanation for this discrepancy.

4) Fortunately, the authors further investigated whether there were differences between the stimulus categories and chose to use a cluster-based approach. Although it was mentioned that the effects persisted over a certain time period, it does not seem obvious how it can be ruled out that this effect is due to the temporal differences in saccade duration and amplitude between categories. It may be necessary to provide a more detailed explanation in the discussion and method section (specifically, the handling of overlapping events) on how the saccade effects were controlled for.

Minor issues:

5) The timing of the saccade onset vs. fixation onset is particularly sensitive to answer the research question, but on page 7, it is mentioned that the authors adjusted the difference in timestamps between the start and end of the recordings to correct for visible linear drifts between EEG and eye-tracking (Unity) timestamps. This seemed to be achieved through a visual inspection and manual editing.

6) It is good that the authors checked the temporal alignment between EEG and eye-tracking data. However, it is not entirely clear how this issue came about and was resolved. Specifically, I would like to know if this adjustment was made to account for some delay due to the eye-tracking (ET) sampling rate (90Hz, which corresponds to ~ 11 ms delay). I wonder if one should be concerned about whether the ET and EEG data is properly aligned. If not, this could have significant implications for the accuracy of the results.

7) Abstract: "Are fixation or saccade onsets a suitable replacement as key events in continuous gaze trajectories (Amme et al., 2024), and can VR effectively capture differences across experimental conditions (Rossion &Jacques, 2008)?": I would suggest removing citations from the abstract if the study is not a direct replication study. In particular, the citation to Amme et al., 2024, which refers to a preprint, seems out of place in the abstract. Additionally, I do not understand what is meant by "differences across experimental conditions".

8) What happened to the ICA weights? Are ICA weights transferred on the unfiltered dataset, the 0.5 Hz filtered dataset or 2Hz filtered dataset?

9) What happened to the IC labelled eye components? Were these rejected (if so, based on what criteria)?

10) The EEG data was down-sampled from 1024 to 500 Hz. This can sometimes lead to artifacts, particularly if the applied filter is not a multiple of 2, which can cause aliasing and distort the signal. What was the rationale behind this?

11) How was the representative subject for the saccade vs. fixation onset study selected? How would the results look when selecting a subject with overall more trials in the head-condition?

12) Figure 4 B and C: it is unclear what data is shown here: is it just the average of all conditions (collapsing head, body and background)? Furthermore, the time windows differ between B and C, what is the time reference here, i.e., how is the P100 defined?

13) The cluster effect starting at the F7 effect seems very early. This is not really explained and might be generally more prone to being noisy (located at the margin and one may see remaining muscle or line noise in Fig. 5D) how much does this effect depend on the inclusion of individual participants/"trials"?

14) Are confidence intervals in plots based on all trials (irrespective of participants) or were conditions first averaged for each participant?

15) Almost 30% of participants were rejected to data issues without further explanation. What can be improved/checked by other researchers that aim for a similar setup?

## Author Response

Reviewer 1 This manuscript aims to provide new insights into suitable methods for analyzing event-related potentials (ERPs) during ecologically valid task performance. Using VR goggles to control biometric signal measurements while creating realistic task scenarios is an intriguing approach. The study addresses a previously unexplored question of whether to compute ERPs based on fixation or saccade events, and has the potential to offer a practical methodology. However, realizing this potential requires providing more detailed explanations of the methodology.

While the manuscript flows well and is easy to read, the simplicity comes at the cost of sacrificing detailed descriptions of the methods. Providing additional methodological details is critical to ensuring reproducibility and scientific rigor.

Please note that this review reflects my perspective as a researcher with expertise in eye movement measurement and autonomic nervous system response acquisition in real-world scenarios but only a general knowledge of EEG.

We thank the reviewer for the positive assessment of our manuscript and hope that we have now sufficiently addressed the mentioned limitations transparently.

Major Comments: ・The visualization for a single participant is a useful approach to outlining the overall narrative (Figures 3-A and B). However, it raises concerns about how this participant was selected, as it might significantly influence the impression of the overall results. Evidence supporting the claim that this participant represents a typical case, or a clear note that this visualization only serves illustrative purposes, should be added. This concern is especially pertinent as the participant in Figure 3C appears to be an outlier compared to others.

Done - We thank the reviewer for pointing this out and acknowledge that the ERP amplitude of our selected subject's head conditions (as seen in the previous Figure 3C) did not represent the broader dataset. In order to provide a more complete overview, we are now showing two additional subjects (updated Figure 3C - F). We have additionally added a remark that the subjects were selected for visualisation purposes:

Page 17: "For visualization purposes, we selected three representative subjects (see Figure 4)" We would also like to point out that in the updated version of the manuscript, the previous Figure 3C - E is no longer included. This was done following the suggestion by Reviewer 2 to make the main message of the manuscript, investigating saccade-onset ERPs and their implications for free-viewing studies, more obvious. For transparency purposes, however, we have included and highlighted our three subjects in the correlation plots below:

Previous Figure 3C - E. (A) - (C) For saccade-onset ERPs, the mean amplitude between 150 - 170 ms of each subject's average ERP is plotted for (A) background vs. body, (B) background vs. head, and (C) body vs. head trials. Each dot represents one subject. The single subject used in the updated Figure 3A and B is highlighted in orange (this is the same subject we had already shown in the previous version of the manuscript). The subject shown in Figure 3C and D is highlighted in green, and the subject shown in Figure 3E and F in blue. The dark-blue lines in the background indicate a (potentially) perfect linear relation between the two conditions. ・The methods used to create Figure 3A require more detailed explanation. Although a prior study is cited (Amme et al., 2024), it would be ideal for the manuscript to stand alone in its methodological clarity. The cited study appears to be a preprint and not yet peer-reviewed, underscoring the need for the methods to be independently validated through this peer-review.

Done - We have now added a more detailed explanation for the method used to create Figure 3A and now 3C and 3E, as seen below:

Page 17: "To compare the difference between fixation- and saccade-onset ERPs, we sorted each subject's fixation-onset trials by the duration of the preceding saccade, in line with Amme and colleagues (2024). The EEG data was aligned and epoched using fixation onsets and then ordered based on saccade durations. Figures 4A, C, and E show the results: fixations onsets are marked by the straight black lines at zero, while saccade onsets are indicated as the preceding curved black lines. If fixation-onsets were the optimal alignment points, we would expect the P100 amplitude peaks to form a straight line 100 ms after fixation onset. However, across all three subjects, the P100 amplitude peaks followed the curved saccade-onset trajectory, suggesting that saccade onsets provide more suitable time points for aligning individual trials in our free-viewing experiment." ・While I acknowledge the difficulty of isolating factors in ecologically valid (noisy) experiments, additional clarification is needed. Is there a possibility that the results are explained by the algorithm that detects saccades and fixations? Specifically: ・Could the detection algorithm's biases impact the results, such as favoring the detection of one type of event (saccade or fixation) over the other? The authors note elsewhere that fewer events lead to higher noise ratios when calculating ERPs. Could the differences in the number of detected events between conditions contribute to the observed effects? ・Some algorithms might not independently detect saccades and fixations, respectively, leading to equal numbers of events. However, even in this case, one type of event (e.g., saccades) might be more "pure," potentially explaining the observed differences in ERP alignment effectiveness. For instance, in such a biased case, fixation events might include fixation and small saccades, whereas saccade events may consist solely of clear saccades, favoring ERP alignment.

Done - We thank the reviewer for pointing out that our trial definition was not sufficiently well described. We now provide more detailed information regarding the eye-tracking algorithm, the detection of events, and the classification of trials. Importantly, the trials are identical for either event onset. In detail, we identified fixations for fixation onsets and used those to cut and align our EEG data, with each fixation resulting in one trial. For saccade onsets, we detected each saccade that preceded a fixation and aligned our data to these events. This means that we have an equal number of trials for either alignment, and the trials themselves are identical but shifted in time, so the saccade-onset trials started on average 60-80 ms before the fixation-onset ones. As a result, if we did not manage to catch individual events, such as small saccades, they would be equally contained in both alignments. We describe the eye-tracking algorithm and the implication for event detection in the text as follows: ● Page 8: "In detail, the continuous eye-tracking data were segmented into smaller intervals (Dar et al., 2021), and a data-driven threshold was calculated for each of these intervals (Keshava et al., 2023; Voloh et al., 2020). Consecutive samples exceeding this threshold were classified as a saccade, and samples below the threshold as fixations. This process resulted in a sequential identification of saccades and fixations throughout the entire recording." ● Page 8: "This approach allowed us to compare identical trials, differing by a time shift: the time-point zero in saccade-onset trials happened several milliseconds before the corresponding time-point of fixation-onset trials. Consequently, if events, such as small saccades and the matching subsequent fixation onsets, were not detected, they would be present in and affect both types of ERPs similarly." Additionally, we investigated whether our algorithm biases the detection of events, for example, favoring one type of event over the other. For this purpose, we inspected the angular eye-velocities surrounding event onsets. If one of the event types, for example, fixations, is less well defined, we would expect that the beginning of fixations sometimes contains samples of the preceding saccade, and therefore, samples of higher velocity. If this were the case, the deviation of velocities around fixation onset would be higher than that of saccade onsets. In contrast, we would not expect to see such a difference should both events be equally well-defined. Investigating our data did not reveal an obvious difference in the variability of velocities. We show the results in Figure 3 and the following accompanying paragraph:

Page 16: "Next, to investigate whether fixation- and saccade-onsets are subject to a bias inherent in the eye movement classification algorithm, we analyzed the distribution of eye movement velocities at event onsets (see Figure 3). Specifically, we examined the variability across trials. If we assume that event onsets are well-defined, we would expect to observe a low variability of velocities around these onsets. In contrast, if the event onsets are not clearly defined, we would anticipate a higher variability. By comparing the variability of velocities at fixation and saccade onsets, we aimed to determine whether our classification algorithm was more precise in defining one type of event over the other. As shown in Figure 3, the velocities at fixation onsets exhibit a relatively low variability within and also across subjects. The distribution of velocities at saccade onsets displays a mirrored distribution, with low variability within and across subjects, exactly one sample before saccade onset. Furthermore, the distributions of data for either event do not overlap. These observations provide support that the classification of fixations and saccades is not influenced by an obvious bias." ・Couldn't fixation-based alignment yield more precise ERP timing? Since saccades are not uniform in duration, could this variability affect the timing accuracy of ERP calculations? Addressed - Investigating saccade-onset ERPs will include the variability of saccade durations, which should influence the results. If saccade-onsets are more accurate triggers, fixation-onsets will lead to a misinterpretation of ERPs, as the timing will not be perfect due to varying saccade durations. This is the central consideration of our study and our findings, and we have added more details to our discussion to make this point more explicit:

Page 30-31: "The selection of saccade-onsets versus fixation-onsets as triggers for ERPs carries significant methodological implications, particularly concerning the timing and interpretation of components like the P100. Aligning ERPs to saccade onset may result in a delayed P100 response compared to classical stimulus-locked ERPs, with the exact timing of this component in relation to saccade events requiring further investigation. Furthermore, the inherent variability in saccade characteristics, such as increased saccade amplitudes over time (Pannasch et al., 2008) or different saccade durations for different stimulus categories, could influence the analysis and interpretation of results. Therefore, investigating whether the methodological shift, from fixation to saccade-onsets, will allow the comparison of neural responses to target categories associated with varying saccade durations is essential." ・If saccade-based alignment improves timing precision, the authors should explain why this is the case.

Done - This is a point we can only speculate on. As saccade-onsets proved to be the relevant triggers for aligning trials, it can be assumed that critical physiological processes affecting ERPs are started at saccade onset. We try to give a potential explanation, but as we are uncertain of the cause, we are careful with the interpretation.

Page 30: "This finding suggests that saccade-onsets serve as more physiologically meaningful triggers for understanding visual processing, marking the initiation of critical neural mechanisms that shape ERP responses (Amme et al., 2024; Gordon et al., 2024; Katz et al., 2020). The underlying processes may be influenced by preparatory activity or predictive planning (Crapse &Sommer, 2008; Katz et al., 2020; Wurtz et al., 2011) or may be guided by attentional shifts (Deubel &Schneider, 1996; Hoffman &Subramaniam, 1995; Kowler et al., 1995; Rolfs et al., 2011), which are linked to enhanced visual discrimination (Deubel &Schneider, 1996). These mechanisms can be associated with the brain preparing for anticipated visual input before initiating the movement (Friston, 2010; Rao &Ballard, 1999), suggesting that early neural activity related to visual selection and saccade preparation might occur before fixation onset (Henderson, 2017). In contrast, fixation-onset triggers might capture neural activity after the initial processing of visual input. As such, utilizing fixation-onsets for self-initiated eye movements could lead to misinterpretations of visual processing timing and dynamics." Minor Comments ・The term "neuronal" is used in several places (e.g., page 2). As ERPs represent the sum of multiple neuronal activities, "neural" might be more appropriate. However, if native speakers find "neuronal" acceptable, this comment can be disregarded (I'm not a native English speaker).

Done - We thank the reviewer for pointing this out. All instances of "neuronal" have been changed to "neural" (Page 3, 7, 31). ・The manuscript could emphasize the advantage of VR goggles with integrated eye trackers in the methods section. While I know this, general readers might not understand which eye tracker was used, given the current description.

Done - We have added an explanation to make this point clear:

Page 5: "The virtual environment was displayed at a constant 90 Hz frame rate via the HTC Vive Pro Eye head-mounted display (HMD; HMD (110{degree sign} field of view, resolution 1440 x 1600 pixels per eye, refresh rate 90 Hz; https://business.vive.com/us/product/vive-pro-eye-office/). The advantage of the HTC Vive Pro Eye HMD is the integrated Tobii eye tracker (0.5{degree sign}-1.1{degree sign} accuracy, 110{degree sign} field of view), allowing us to actively record the subject's eye movements." ・In Figure 3B, "gaze" might be better replaced with "fixation" to avoid confusion.

Done - We have replaced "gaze" with "fixation" in Figure 4B,D,F (previous Figure 3B) and additionally in Figure 5A and Figure 2B-C where we noticed the same oversight. ・On page 8, there is a notation "(s." which appears to be a typographical error. Please correct this.

Done - This was indeed a typographical error, meant to be "see". It has been corrected.

Reviewer 2 The present study investigated neural responses to heads and bodies of virtual agents compared to other visual objects in a free-viewing virtual reality environment. Here, the authors focused on advancing methodological considerations when facing typical challenges of investigating face processing in a naturalistic setup compared to controlled static setups. Although the initial objective of replicating the classic N170 face effect in the VR environment was not achieved, the results revealed significant category differences in the processing of heads, bodies, and other visual objects during the P100 and N170 time windows when using a cluster-based approach. The use of co-registered EEG and eye-tracking data allowed the authors to investigate the impact of saccade-onsets on ERP analysis. The results showed that ERPs based on saccade-onsets yielded clearer and more well-defined early brain responses compared to fixation onsets, consistent with recent findings from a MEG study by Amme et al. (2024).

The authors address a timely and relevant topic by investigating face processing in a more naturalistic setting, moving away from the traditional controlled laboratory environment. Their approach of using virtual reality (VR) to achieve better controllability and standardization is well-justified. Also, the authors have successfully presented their methodology and approach in a transparent and clear way, making the manuscript easy to read and to the point. The decision to preview the results at the beginning of the manuscript is unconventional, but in this case, it is effective in setting the reader's expectations, as the actual results diverge from the hypothesized outcomes. Finally, the authors demonstrate transparency by acknowledging potential limitations of the study which adds to the overall quality of the manuscript.

We thank the reviewer for this positive evaluation of the current manuscript and the insightful comments. We hope our revisions address all points in a transparent manner.

My main difficulties with the manuscript lie in the fact that it is not entirely clear whether the methodological aspect is the primary focus (saccade-onset vs. fixation-onset) or if the content is actually about face perception (or if it is only used as an application case). In either case, I feel a more detailed elaboration of the theoretical aspects and implications is missing: a) In the first case, where the methodological aspect is the primary focus, I would have expected a discussion of the analytical approach, not only including one selected participant but the distribution of these patterns across the whole sample, discussion about the implications of using saccade-onset or fixation-onset as triggers for ERPs, particularly in relation to predictions (saccade planning), movement, and timing, and an investigation about the expected differences in processing windows compared to fixed stimulus presentations. b) In the second case, where face perception is the primary focus, I would have liked to see the authors attempt to provide additional explanations for their results, particularly because the typical N170 face effect was not found. The authors themselves mention some possible reasons, but they do not explore or address these further to better understand the results:

Addressed - We sincerely appreciate the reviewer's feedback and the opportunity to clarify our study's main message. We aim to explore the methodological challenges of using VR to investigate neural processes under natural conditions, particularly the considerations that arise with this approach. Our primary interest is in comparing saccade versus fixation-onset ERPs in a free-viewing VR study, examining condition-specific differences to better understand the impact of aligning trials to saccade onsets. Since different stimulus categories can be linked to varying saccade amplitudes, this approach underscores the importance of saccade-onset investigations.

Face perception and the N170 component are well-defined and understood, so our study uses them as examples. While our findings contribute to face perception research, their primary role is to demonstrate the methodological implications of using saccade onsets versus fixation onsets as event markers.

To ensure our central message is clearer, we have significantly revised the manuscript, specifically the abstract, introduction, and discussion, incorporating the reviewer's suggestions and recommendations. We list several adjustments of the main text, specifically focussed on a) below:

Abstract: ● Page 1: "[...] and consequently, can VR capture differences across different stimulus categories associated with varying saccade durations?" ● Page 1: "In summary, employing VR, EEG, and eye-tracking to investigate differences across fixation categories provides insights into the relevance of saccadic onsets as event triggers and enhances our understanding of cognitive processes in naturalistic settings." Introduction: ● Page 3: "Building on these findings, a question arises: Do saccade onsets provide the optimal alignment for ERP analysis in free-viewing studies? Furthermore, if saccade-onsets are the preferred alignment, can VR capture differences across stimulus categories varying in saccade characteristics? To address the first question, we can assess the timing of a saccade-onset P100 in an immersive free-viewing study. Employing these saccade-onset ERPs to study a well-established effect, such as the N170 face effect (Eimer, 2011; Rossion &Jacques, 2008), can tackle the second question." Discussion: ● Page 30: "This finding suggests that saccade-onsets serve as more physiologically meaningful triggers for understanding visual processing, marking the initiation of critical neural mechanisms that shape ERP responses (Amme et al., 2024; Gordon et al., 2024; Katz et al., 2020). The underlying processes may be influenced by preparatory activity or predictive planning (Crapse &Sommer, 2008; Katz et al., 2020; Wurtz et al., 2011) or may be guided by attentional shifts (Deubel &Schneider, 1996; Hoffman &Subramaniam, 1995; Kowler et al., 1995; Rolfs et al., 2011), which are linked to enhanced visual discrimination (Deubel &Schneider, 1996). These mechanisms can be associated with the brain preparing for anticipated visual input before initiating the movement (Friston, 2010; Rao &Ballard, 1999), suggesting that early neural activity related to visual selection and saccade preparation might occur before fixation onset (Henderson, 2017). In contrast, fixation-onset triggers might capture neural activity after the initial processing of visual input. As such, utilizing fixation-onsets for self-initiated eye movements could lead to misinterpretations of visual processing timing and dynamics." ● Page 30-31: "The selection of saccade-onsets versus fixation-onsets as triggers for ERPs carries significant methodological implications, particularly concerning the timing and interpretation of components like the P100. Aligning ERPs to saccade onset may result in a delayed P100 response compared to classical stimulus-locked ERPs, with the exact timing of this component in relation to saccade events requiring further investigation. Furthermore, the inherent variability in saccade characteristics, such as increased saccade amplitudes over time (Pannasch et al., 2008) or different saccade durations for different stimulus categories, could influence the analysis and interpretation of results. Therefore, investigating whether the methodological shift, from fixation to saccade-onsets, will allow the comparison of neural responses to target categories associated with varying saccade durations is essential." Furthermore, as we believe that face perception provides a relevant piece to the overall story, we have additionally followed the reviewer's recommendation regarding b) and provided detailed responses in the sections below.

1) It is mentioned that there may not be enough trials in which participants were looking at the head, and that there may have been a lot of variation due to the head orientation of the avatars and movement. While it may be inconvenient, it would be useful to know how often faces (seen relatively frontally from a rather close distance) were actually viewed. At least as a manipulation check, it would be good to see how the reaction to frontal faces was. If this is not possible, I wonder if this paradigm is suitable for the investigation of neural face processing (if faces were hardly viewed at all). Perhaps the controlled VR setup could be used for a different research question and a more specific focus on saccade-onset vs. fixation-onsets could be explored.

Done - First, to investigate distances, we specified in the Methods section that in our city one unity unit corresponds to one meter: "The dimensions of the virtual city matched the real world, with one unity unit corresponding to one meter, allowing us to indicate distances using meters." (Page 7).

Using this information, we investigated the viewing angle and the distances kept while viewing pedestrians. We also investigated whether frontal faces and faces viewed at close distances affect the ERP curves. The results can be seen in Figure 6. While we aggregated over all head trials, most of these corresponded to relatively frontal face fixations and, therefore, investigating only frontal faces differed little from heads. Investigating frontal faces at close distances reduced the number of trials drastically and made the interpretation of results difficult. Nonetheless, we believe the overall curve of frontal and close faces follows that of all heads. As a result, we believe that it is justifiable to aggregate over all head trials. We have summarised our results as follows:

Page 24: "Before investigating the presence of an N170 effect, typically associated with the perception of faces (Eimer, 2011; Rossion &Jacques, 2008), we assessed that aggregating all fixations on heads, irrespective of viewing angles, does not obscure any potential differences. To this end, inspecting the viewing angle distribution across participants (see Figure 6A) revealed that most fixations are directed toward pedestrians' faces. Specifically, computing the circular mean within participants, followed by the circular mean and the circular standard deviation across participants, resulted in average viewing angles of 13.210 {plus minus} 30.868{degree sign}. Additionally, to exclude viewing distance as a potential influence, we computed the median and MAD across participants (5.209 {plus minus} 2.591 m; see Figure 6B), indicating that most heads are viewed at a close range. When we investigated the ERPs from fixations on heads (151 {plus minus} 133 trials) compared to those on only faces (72 {plus minus} 64 trials), no visible differences other than a slight increase in noise levels emerged, suggesting that aggregating all head fixations is valid. Only inspecting frontal faces viewed at a close distance (less than 5 m) resulted in fewer trials (39 {plus minus} 38 trials) and an ERP with high noise levels, making the ERP curve challenging to interpret. Overall, our results indicate no discernible differences elicited by viewing angles of distances, confirming that aggregating head fixations is appropriate." 2) The effectiveness of the stimulus material is not addressed. Was this material previously validated (internally or externally)? Would the avatars be able to evoke the expected effects in a non-VR or static setup? Are the avatars perceived as aversive (uncanny valley), leading to avoidance of fixation? Perhaps a specific task for the participants could help ensure that they are viewing the faces, such as "find person X". Additionally, I wonder if the authors would advise in favor or against using artificial avatars (in comparison to video recordings of real faces) in future studies.

Addressed - We had not previously validated our stimulus material, but we agree that this is an important aspect. To rectify this oversight, we designed a survey using images of our virtual avatars and other, previously published stimuli (unrealistic, semi-realistic, realistic face stimuli; Gorlini et al., 2023), assessing the possibility of an uncanny valley effect via the indices humanness, eeriness, and attractiveness (Ho &MacDorman, 2010). For the indices of humanness and eeriness, our avatars most closely resembled the ratings for the unrealistic faces. Only for attractiveness did our stimuli differ from the unrealistic faces. As humanness and eeriness are the most commonly used indices, we believe that our data does not elicit an uncanny valley effect. We included Figure 9, visualizing these results, and describe them as follows:

Methods - Page 11-12: "To validate our stimuli, we collected a separate dataset investigating the potential presence of an uncanny valley effect. Twelve participants (eight female, zero diverse; mean age 31.615 {plus minus} 13.301) completed an online survey. Among the participants, two were very familiar with virtual avatars, four were familiar, four were neutral, one was unfamiliar, and one was very unfamiliar. We randomly selected ten images taken from our avatars and compared them to stimuli from the study by Gorlini et al. (2023). The stimuli from Gorlini et al. (2023) were categorized into three types: unrealistic, semi-realistic, and realistic faces. Ten stimuli were randomly selected from each category, resulting in 40 images, including our virtual avatars. All images were standardized in size and resolution and displayed against a grey background. Participants rated the images using a survey developed by Ho and MacDorman (2010) to investigate the presence of an uncanny valley effect. This survey evaluates images based on three indices: humanness (six items), eeriness (eight items), and attractiveness (four items), with semantic differential items assessed using a five-point Likert scale. For all stimulus categories (VR avatars, unrealistic, semi-realistic, realistic), we calculated average scores for each participant for each index. Statistical differences were evaluated using a separate Friedman test per index." Results - Page 28-29: "To assess whether our virtual avatars elicit an uncanny valley effect, we analyzed survey responses, comparing our avatars to other unrealistic, semi-realistic, and realistic face images. If our avatars induced this effect, we would expect them to show a higher humanness than the unrealistic faces and an increased eeriness compared to unrealistic or realistic faces, i.e., behaving most similar to the semi-realistic faces, reported to induce an uncanny valley effect (Gorlini et al., 2023). Inspecting our results revealed semi-realistic avatars to exhibit this pattern, while our avatars resembled the unrealistic ones (see Figure 9). A Friedman test confirmed significant differences between stimulus categories for humanness, ꭓ²(3) = 24.3, p < 0.001, eeriness, ꭓ²(3) = 15.4, p = 0.001, and attractiveness, ꭓ²(3) = 27.5, p < 0.001. Post-hoc tests revealed significant differences, particularly between the realistic category and all others for humanness, between semi-realistic and realistic for eeriness, and for attractiveness between VR avatars and the realistic and unrealistic categories, as well as between the semi-realistic category and the realistic group. While our avatars show differences from the unrealistic category in attractiveness, they aligned most closely with the unrealistic category for humanness and eeriness. These findings suggest that our avatars do not strongly induce an uncanny valley effect." Based on our results, we would advise using similar stimuli in future studies if appropriate. However, while increasing realism in future stimulus sets might enhance ecological validity, the presence of an uncanny valley effect will also become more likely. Therefore, similar assessments to the one presented above are highly advisable for future studies. We describe this in the text as follows:

Page 29: "In contrast, increasing realism will also increase the possibility of an uncanny valley effect of virtual agents (Schindler et al., 2017), something future studies should carefully examine. Nonetheless, we recommend using artificial avatars, as they offer a high degree of interaction, modification (Schindler et al., 2017), or specific positioning (Sánchez Pacheco et al., 2025) compared to static images or video recordings." 3) The authors do not provide a clear explanation for the findings, aside from the increased noise level. They cite a study by Gert et al. 2022, showing that a fixation-related N170 ERP has been found in previous free-viewing setups, including those with complex scenes. However, this is not the case in the current study. I would have expected the authors to provide a more detailed explanation for this discrepancy.

Done - We now provide a more detailed explanation for the absence of an N170 effect. Taken together, we are not certain about the underlying cause, but we try to give more possible explanations based on recent literature and our own study as follows:

Page 33: "Several factors may explain the absence of an effect in this study. First, many free-viewing studies show smaller ERP components compared to stimulus-locked studies (Auerbach-Asch et al., 2020; Gert et al., 2022; Ladouce et al., 2022), a difference that may be even more pronounced in VR-based research. Second, methodological aspects could play a role. While virtual faces have been shown to elicit an N170 effect, it is typically weaker than in two-dimensional studies (Kirasirova et al., 2025). To our knowledge, our study is the first to examine face perception in a free-viewing, free-movement VR setup, introducing new variables such as attentional shifts or stimulus variability in a virtual environment, which may contribute to the absent effect. Additionally, other methodological factors, such as the virtual environment or the density of visual stimuli, may contribute to the observed findings. Third, individual differences and hemispheric variations in the N170 response (De Vos et al., 2012) could introduce variability, rendering an across-subject investigation difficult. Overall, determining the exact cause of the absence of an N170 face effect remains complex and warrants future investigation." 4) Fortunately, the authors further investigated whether there were differences between the stimulus categories and chose to use a cluster-based approach. Although it was mentioned that the effects persisted over a certain time period, it does not seem obvious how it can be ruled out that this effect is due to the temporal differences in saccade duration and amplitude between categories. It may be necessary to provide a more detailed explanation in the discussion and method section (specifically, the handling of overlapping events) on how the saccade effects were controlled for.

Addressed - We have adjusted our Methods section and the Discussion to provide more details regarding this point. Specifically, as we investigated saccade-onset ERPs, we chose not to model saccade amplitudes. In principle, supplying saccade amplitudes and the highly correlated saccadic durations to Unfold would allow Unfold to mimic the effect. However, if saccade durations explain most of the variance, we have the simplest explanation available. As a result, with this first investigation, we did not want to mix complicated models. However, we do acknowledge that we cannot rule out potential effects of saccade amplitudes on our observed results. We changed the phrasing of the discussion to be more careful regarding this point. The changes made to the paper are as follows:

Methods - Page 10-11: "Specifically, to investigate differences between fixation categories, we implemented the linear model to correct for overlapping events, indicated by saccade onsets. As we investigate differences in saccade-onset ERPs, we did not model saccade amplitudes due to their high correlation with saccade durations (Guadron et al., 2022; Harris &Wolpert, 2006)." Discussion - Page 32: "Relatedly, was our decision not to model saccade amplitudes despite their known influence on the P100 effect (Ehinger &Dimigen, 2019; Guadron et al., 2022; Henderson et al., 2013). Instead, we investigated differences in saccade-onset ERPs, aligning neural responses across categories while accounting for saccade durations, which strongly correlate with saccade amplitudes (Guadron et al., 2022; Harris &Wolpert, 2006). Given this correlation, we believe our approach did sufficiently control for saccade effects. However, further testing should rule out the saccade amplitudes' contribution to the observed face effects. Here, a study contrasting categories with comparable amounts of trials would be essential." Minor issues:

5) The timing of the saccade onset vs. fixation onset is particularly sensitive to answer the research question, but on page 7, it is mentioned that the authors adjusted the difference in timestamps between the start and end of the recordings to correct for visible linear drifts between EEG and eye-tracking (Unity) timestamps. This seemed to be achieved through a visual inspection and manual editing.

Done: We respond to this point together with point 6 (see the response below).

6) It is good that the authors checked the temporal alignment between EEG and eye-tracking data. However, it is not entirely clear how this issue came about and was resolved. Specifically, I would like to know if this adjustment was made to account for some delay due to the eye-tracking (ET) sampling rate (90Hz, which corresponds to ~ 11 ms delay). I wonder if one should be concerned about whether the ET and EEG data is properly aligned. If not, this could have significant implications for the accuracy of the results.

Addressed - We thank the reviewer for this comment and acknowledge that our explanation of this point was unclear. During EEG preprocessing, we observed a linear drift between the Unity and EEG time streams over the course of the recording. This was not related to the difference in sampling rates. We are not completely certain of the exact cause of this, but based on extensive testing, we assume it is the result of an internal delay within Unity that LSL cannot access. To address this issue, we performed a global drift correction by using a single correction factor that was linearly added to all timestamps of the eye-tracking data. This adjustment successfully aligned the data, eliminating visible drifts. Importantly, an identical correction was applied to fixation and saccade onset conditions. As a result, comparing the timing of the saccade vs. fixation onsets should remain unaffected by this. However, we 100% agree with the reviewer that the timing is a central aspect of the analysis. Due to this, we were very conservative when excluding recordings and only kept those where we were certain the EEG and ET streams were properly aligned. This exclusion was also partially responsible for the high amount of excluded data (see the response to comment 15). We have now clarified this in the manuscript as follows:

Page 8-9: "Aligning our data worked; however, visual inspection indicated a small constant linear drift between the overall EEG and eye-tracking (Unity) timelines. To correct this drift, we calculated the difference between the first EEG and eye-tracking timestamps and between the last ones, computed the deviation between these differences, and applied it linearly to the eye-tracking timeline. Twenty-one subjects displayed a more substantial drift, requiring the start-end deviation up to four times or to adjust the timeline by one (11 ms) or two (22 ms) sample(s), according to the 90 Hz sampling rate, over the course of a 30-minute experimental session. Notably, this drift correction was identical for fixation and saccade onsets. The final dataset only included subjects for which we were confident in aligning the two data streams (also see Methods - Subject)." Additionally, we give recommendations in the discussion for other researchers to avoid such an issue:

Page 34: "One of them, the alignment of various data streams, could be tackled by employing state-of-the-art amplifiers with a built-in connection or direct support for data synchronization tools such as LSL to improve the temporal accuracy of recordings further (Gramann, 2024). In this sense, effective and reliable synchronization would reduce alignment or drift issues that led to the exclusion of subjects' data in the present study." 7) Abstract: "Are fixation or saccade onsets a suitable replacement as key events in continuous gaze trajectories (Amme et al., 2024), and can VR effectively capture differences across experimental conditions (Rossion &Jacques, 2008)?": I would suggest removing citations from the abstract if the study is not a direct replication study. In particular, the citation to Amme et al., 2024, which refers to a preprint, seems out of place in the abstract. Additionally, I do not understand what is meant by "differences across experimental conditions".

Done - The "experimental conditions" phasing was replaced by "stimulus categories". With this, we describe heads, bodies, and background stimuli. We have removed (Rossion &Jacques, 2008) from the abstract.

Discussed - We have decided to keep the Amme et al., 2024 citation in the abstract given its instrumental role and inspiration in analyzing the present, and we wish to honor it appropriately. However, if the reviewer or editor prefers its removal, we are happy to accommodate.

8) What happened to the ICA weights? Are ICA weights transferred on the unfiltered dataset, the 0.5 Hz filtered dataset or 2Hz filtered dataset? Done - We have further explained how the ICA weights were used during the preprocessing as follows:

Page 10: "ICA weights were then transferred to the dataset filtered at 0.5 Hz." 9) What happened to the IC labelled eye components? Were these rejected (if so, based on what criteria)? Done - We have specified the components identified by IC label, provided statistics, and indicated the rejection protocol:

Page 10: "Components containing more than 80% muscle activity (mean = 16 components; std = 7.407) or more than 90% of other noise (ocular movement, mean = 2.121, std = 0.331; channel noise, mean = 0.303, std = 0.529; cardiac artifact, mean = 0.060, std = 0.242; line noise, mean = 0.030, std = 0.174), as identified by ICLabel (Pion-Tonachini et al., 2019), were removed automatically." 10) The EEG data was down-sampled from 1024 to 500 Hz. This can sometimes lead to artifacts, particularly if the applied filter is not a multiple of 2, which can cause aliasing and distort the signal. What was the rationale behind this? Addressed - We implemented the downsampling procedure from 1024 to 500 Hz since it is recommended by Klug and Kloosterman (2022) for an adequate implementation of the Zapline Plus plugging to remove spectral peaks around 50 Hz (line noise) and, separately, 90 Hz (noise from VR glasses). Furthermore, before the downsampling, we added a low-pass filter to the data (128 Hz) to prevent any issues with the downsampling procedure. We have made this point more visible and understandable in our method section:

Page 9: "Following the recommendation of Klug and Kloosterman (2022), we downsampled the EEG data from 1024 Hz to 500 Hz to apply a line noise filter from the 'zapline plus' plugin (Klug &Kloosterman, 2022, based on de Cheveigné, 2020). We conducted this procedure to automatically remove spectral peaks around 50 Hz and, separately, 90 Hz." 11) How was the representative subject for the saccade vs. fixation onset study selected? How would the results look when selecting a subject with overall more trials in the head-condition? Done - We originally selected a representative subject we believed would demonstrate the effect efficiently. In this updated version, we have now added two additional subjects to demonstrate the effect for more than one subject. This includes one subject with 361 head trials, shown in Figure 3E and 3F. Overall, while individual differences are visible, we believe the preferred timing of saccade-onsets is visible in all three subjects.

12) Figure 4 B and C: it is unclear what data is shown here: is it just the average of all conditions (collapsing head, body and background)? Furthermore, the time windows differ between B and C, what is the time reference here, i.e., how is the P100 defined? Done - Indeed, we show the average across all trials, irrespective of the condition. We have updated the figure's caption (now Figure 5) to make this point clear:

Page 23: "(B) and (C) Topoplots across all trials irrespective of the stimulus category (the average of all background, body, and head trials)" Furthermore, we have included an explanation regarding the differences in the time windows in 5B and 5C. We only selected temporal windows for visualization based on the ERPs' visual inspection. Furthermore, we aimed to maintain a constant shift of 60 ms between fixation and saccade onsets for all three topoplots:

Page 23: "For visualization purposes, we selected time intervals based on the visual inspection of fixation- and saccade-onset ERPs, maintaining a constant difference between fixation- and saccade-onsets" 13) The cluster effect starting at the F7 effect seems very early. This is not really explained and might be generally more prone to being noisy (located at the margin and one may see remaining muscle or line noise in Fig. 5D) how much does this effect depend on the inclusion of individual participants/"trials"? Addressed - We observe the cluster peak very early, close to the cluster's margin, which makes it attractive to speculate about underlying contributions, such as the noisiness of the signal. If this is the case, we cannot rule it out in the current investigation. However, the precise boundaries of a cluster also depend on the interior, meaning, these boundaries must be interpreted with caution. Furthermore, we believe that if our results were to depend on only a small number of subjects or trials, this would create huge error bars, and nothing should be significant, but we acknowledge that we cannot test this currently. Instead, we now acknowledge this remark in our manuscript: ● Page 27: "Of note, while this cluster effect starts quite early, it does not reach negative times." ● Page 32: "First, the observed face effect may be influenced by residual noise, particularly given that the cluster peak is located near the cluster's margin. While we aimed to minimize these factors through preprocessing, individual participant or trial variability may still contribute to the effect." Additionally, thanks to your comment, we revised the reporting of the TFCE results. In the previous version, we only reported the largest cluster, which we thought was the most appropriate approach as we wanted to avoid a multiple-comparison issue. However, we do not want to exclude the information about other significant clusters, so we now included them in as extended data (Table 8-1). We thank the reviewer for making us aware of this oversight.

14) Are confidence intervals in plots based on all trials (irrespective of participants) or were conditions first averaged for each participant? Done - Due to the different number of trials per participant, we first averaged within participants (all trials) and then took the average across participants. The confidence intervals in Figure 7 (previously Figure 5) correspond to the average across participants. We now indicate this in the figure caption and also explicitly mention it when we first talk about the average across participants: ● Page 26: "[...] indicating the average across participants" ● Page 21: "For this, we first averaged within subjects to account for the high variability of head trials and then averaged across subjects."
