# Supplementary material for: Investigating Saccade-Onset Locked EEG Signatures of Face Perception during Free-Viewing in a Naturalistic Virtual Environment
Source: eNeuro. 2025 Aug 29;12(9):ENEURO.0573-24.2025. doi: 10.1523/ENEURO.0573-24.2025 (PMC12418071; doi:10.1523/ENEURO.0573-24.2025)
Supplement: Figure 8-1 — Description of additional clusters found using TFCE. Download Figure 8-1, DOCX file. [file eneuro-12-ENEURO.0573-24.2025-s003.docx]

Figure 8-1. Description of additional clusters found using TFCE.

| Peak (time) | Peak (channel) | F statistic | *p* value | Spatial extension | Temporal extension |
| --- | --- | --- | --- | --- | --- |
| *Post-stimulus onset* | | | | | |
| 374 ms | F1 | 15.31 | 0.0173 | 8 electrodes | 372 to 378 ms |
| 272 ms | AF4 | 16.04 | 0.0035 | 27 electrodes | 266 to 290 ms |
| 272 ms | CP3 | 13.61 | 0.0039 | 26 electrodes | 258 to 310 ms |
| 838 ms | C1 | 14.88 | 0.0210 | 6 electrodes | 832 to 844 ms |
| 36 ms | FP1 | 12.26 | 0.0141 | 25 electrodes | 12 to 40 ms |
| *Pre-stimulus onset* | | | | | |
| -280 ms | FCz | 16.52 | 0.0107 | 5 electrodes | -292 to -278 ms |
| -396 ms | TP7 | 10.51 | 0.0436 | 3 electrodes | -398 to -394 ms |
| -396 ms | FC2 | 22.94 | 0.0003 | 47 electrodes | -402 to -342 ms |
| -406 ms | T7 | 13.42 | 0.0262 | 5 electrodes | -408 to -404 ms |
| -408 ms | AF7 | 11.81 | 0.0353 | 2 electrodes | -408 to -404 ms |
