# Supplementary material for: Investigating Saccade-Onset Locked EEG Signatures of Face Perception during Free-Viewing in a Naturalistic Virtual Environment
Source: eNeuro. 2025 Aug 29;12(9):ENEURO.0573-24.2025. doi: 10.1523/ENEURO.0573-24.2025 (PMC12418071; doi:10.1523/ENEURO.0573-24.2025)
Supplement: Figure 9-1 — Average ratings of the different picture categories on three indices. Download Figure 9-1, DOCX file. [file eneuro-12-ENEURO.0573-24.2025-s004.docx]

Figure 9-1. Average ratings of the different picture categories on three indices.

| Stimuli | Attractiveness  (Mean, STD) | Humanness  (Mean, STD) | Eeriness  (Mean; STD) |
| --- | --- | --- | --- |
| VR Avatars | 2.56 (0.76) | 2.01 (0.71) | 2.75 (0.70) |
| Realistics | 3.59 (0.55) | 4.66 (0.39) | 2.50 (0.56) |
| Semi-Realistics | 2.72 (0.58) | 2.14 (0.71) | 3.36 (0.64) |
| Unrealistics | 3.02 (0.44) | 1.89 (0.74) | 2.98 (0.70) |
