# Supplementary material for: Investigating Saccade-Onset Locked EEG Signatures of Face Perception during Free-Viewing in a Naturalistic Virtual Environment
Source: eNeuro. 2025 Aug 29;12(9):ENEURO.0573-24.2025. doi: 10.1523/ENEURO.0573-24.2025 (PMC12418071; doi:10.1523/ENEURO.0573-24.2025)
Supplement: Figure 9-2 — Results of the Friedman tests and post-hoc analysis for the three indices. Download Figure 9-2, DOCX file. [file eneuro-12-ENEURO.0573-24.2025-s005.docx]

Figure 9-2. Results of the Friedman tests and post-hoc analysis for the three indices.

| Subscale | ꭓ²(3) | *p* value | Difference direction |
| --- | --- | --- | --- |
| Attractiveness | 27.50 | 4.6245e-06 | Mean rank for VR Avatars stimuli is significantly different from Realistics and Unrealistics stimuli.  Mean rank for Semi Realistics stimuli is significantly different than Realistics stimuli. |
| Humanness | 24.30 | 2.1624e-05 | Mean rank for Realistics stimuli is significantly different from VR Avatars, Semi Realistics, and Unrealistics stimuli. |
| Eeriness | 15.40 | 0.0015 | Mean rank for Realistic stimuli is significantly different from Semi-Realistic stimuli. |
